# Novel hormone therapies for advanced prostate cancer: Understanding and countering drug resistance

**DOI:** 10.1016/j.jpha.2025.101232

**Published:** 2025-02-22

**Authors:** Zhipeng Wang, Jie Wang, Dengxiong Li, Ruicheng Wu, Jianlin Huang, Luxia Ye, Zhouting Tuo, Qingxin Yu, Fanglin Shao, Dilinaer Wusiman, William C. Cho, Siang Boon Koh, Wei Xiong, Dechao Feng

**Affiliations:** a Department of Urology, Sichuan Provincial People's Hospital, University of Electronic Science and Technology of China, Chengdu, 610072, China; b Department of Urology, Institute of Urology, West China Hospital, Sichuan University, Chengdu, 610041, China; c Department of Public Research Platform, Taizhou Hospital of Zhejiang Province Affiliated to Wenzhou Medical University, Taizhou, Zhejiang, 317000, China; d Department of Urological Surgery, Daping Hospital, Army Medical Center of PLA, Army Medical University, Chongqing, 400042, China; e Department of Pathology, Ningbo Clinical Pathology Diagnosis Center, Ningbo, Zhejiang, 315211, China; f Department of Rehabilitation, The Affiliated Hospital of Southwest Medical University, Luzhou, Zhejiang, 646000, China; g Department of Comparative Pathobiology, College of Veterinary Medicine, Purdue University, West Lafayette, IN, 47907, USA; h Purdue Institute for Cancer Research, Purdue University, West Lafayette, IN, 47907, USA; i Department of Clinical Oncology, Queen Elizabeth Hospital, Hong Kong SAR, 999077, China; j Faculty of Health and Life Sciences, University of Bristol, Bristol, BS8 1TD, UK; k Division of Surgery & Interventional Science, University College London, London W1W 7TS, UK

**Keywords:** Novel hormone therapies, Advanced prostate cancer, Drug resistance, Androgen receptor

## Abstract

Prostate cancer is the most prevalent malignant tumor among men, ranking first in incidence and second in mortality globally. Novel hormone therapies (NHT) targeting the androgen receptor (AR) pathway have become the standard of care for metastatic prostate cancer. This review offers a comprehensive overview of NHT, including abiraterone, enzalutamide, apalutamide, darolutamide, and rezvilutamide, which have demonstrated efficacy in delaying disease progression and improving patient survival and quality of life. Nevertheless, resistance to NHT remains a critical challenge. The mechanisms underlying resistance are complex, involving *AR* gene amplification, mutations, splice variants, increased intratumoral androgens, and AR-independent pathways such as the glucocorticoid receptor, neuroendocrine differentiation, DNA repair defects, autophagy, immune evasion, and activation of alternative signaling pathways. This review discusses these resistance mechanisms and examines strategies to counteract them, including sequential treatment with novel AR-targeted drugs, chemotherapy, poly ADP-ribose polymerase inhibitors, radionuclide therapy, bipolar androgen therapy, and approaches targeting specific resistance pathways. Future research should prioritize elucidating the molecular basis of NHT resistance, optimizing existing therapeutic strategies, and developing more effective combination regimens. Additionally, advanced sequencing technologies and resistance research models should be leveraged to identify novel therapeutic targets and improve drug delivery efficiencies. These advancements hold the potential to overcome NHT resistance and significantly enhance the management and prognosis of patients with advanced prostate cancer.

## Highlights

• A comprehensive review of NHT for prostate cancer.
• A detailed examination of NHT resistance mechanisms and innovative strategies to overcome these resistances.
• Future research directions regarding NHT resistance.

## Graphical abstract

**Figure:**
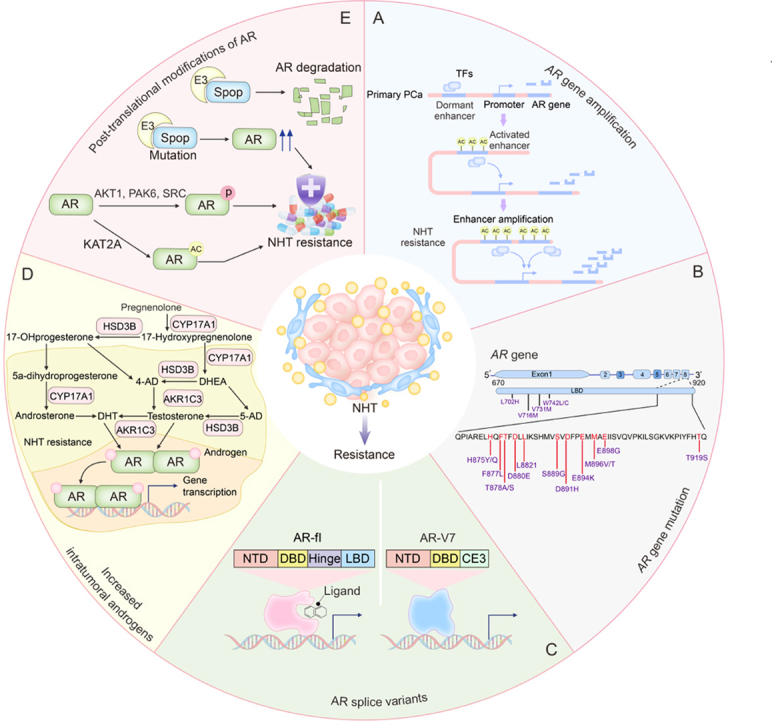

## 1 Introduction

Prostate cancer is the most prevalent malignant solid tumor among men globally, ranking first in incidence and second in mortality [1]. The prevalence of prostate cancer at various stages shows substantial regional variation. In western countries, metastatic cases comprise approximately 5%–8% of all prostate cancer diagnoses. However, in regions with limited access to early screening, such as many Asian countries, metastatic cases account for 25%–44% of diagnoses [2].

Although radical prostatectomy and radiotherapy are effective for treating early localized prostate cancer, approximately 30%–47% of such patients experience recurrence, radiotherapy resistance, or metastasis, ultimately succumbing to the disease [3,4]. For advanced prostate cancer, the androgen receptor (AR) pathway plays a critical role in tumor cell survival, establishing endocrine therapies targeting this pathway as the cornerstone of treatment. Historically, standard endocrine therapies combined androgen deprivation therapy, either medical or surgical, with first-generation anti-androgens such as bicalutamide and flutamide. However, with the advent of more potent novel hormone therapies (NHT), these first-generation anti-androgens have become insufficient for managing contemporary prostate cancer, even in hormone-sensitive stages. The NHT, including abiraterone, enzalutamide, apalutamide, darolutamide, and rezvilutamide, represent significant advancements over first-generation anti-androgens, effectively delaying disease progression and improving survival and quality of life. Despite these advancements, resistance to NHT remains inevitable, posing a substantial barrier to further improving patient outcomes.

This review systematically examines the role of NHT in the treatment of advanced prostate cancer, summarizes the mechanisms contributing to therapy resistance, and explores potential strategies to overcome these challenges.

## 2 Introduction to NHT drugs

**Fig. 1:**
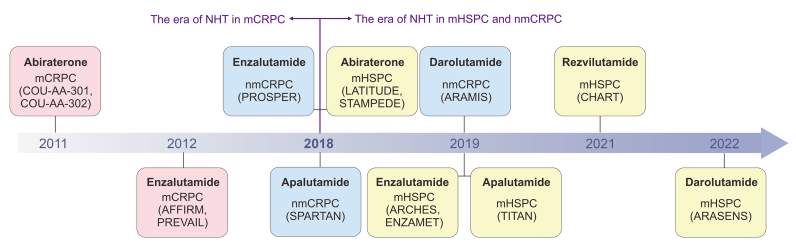
Timeline of approval and indications for novel hormone therapies (NHT) in prostate cancer. This figure illustrates the timeline of the approval and indications for various NHT used in the treatment of advanced prostate cancer. Starting from 2011, the timeline includes key NHT drugs such as abiraterone, enzalutamide, apalutamide, darolutamide, and rezvilutamide. Key clinical trials that supported the approval of these therapies are also indicated. mCRPC: metastatic castration-resistant prostate cancer; mHSPC: metastatic hormone-sensitive prostate cancer; nmCRPC: non-metastatic castration-resistant prostate cancer.

In recent decades, the development of NHT drugs has advanced significantly. Results from numerous Phase III randomized controlled trials (RCTs) have expanded the indications for these drugs to include multiple advanced stages of prostate cancer. These stages encompass metastatic castration-resistant prostate cancer (mCRPC), metastatic hormone-sensitive prostate cancer (mHSPC), and non-metastatic castration-resistant prostate cancer (nmCRPC). This section provides a comprehensive review of NHT drug applications, charting their developmental progress as demonstrated by these pivotal RCTs (Fig. 1, and Tables 1 and S1). Detailed content of this section is included in Supplementary material.

**Table 1:** Phase III randomized clinical trials of novel hormone therapy.

Study	ID	Treatments	Disease stage	Number of patients	Primary outcomes
COU-AA-301	NCT00638690	Abiraterone vs. Placebo	mCRPC	1195	OS: 15.8-mo vs. 11.2-mo (HR: 0.74; 95% CI: 0.64–0.86; *P* < 0.001) rPFS: 5.6-mo vs. 3.6-mo (HR: 0.66; 95% CI: 0.58–0.76; *P* < 0.001)
COU-AA-302	NCT00887198	Abiraterone vs. Placebo	mCRPC	1088	OS: 34.7-mo vs. 30.3-mo (HR: 0.81; 95% CI: 0.70–0.93; *P* = 0.003) rPFS: 16.5-mo vs. 8.2-mo (HR: 0.52; 95% CI: 0.45–0.61; *P* < 0.001)
LATITUDE	NCT01715285	Abiraterone vs. Placebo	High-risk mHSPC^a^	1199	OS: 53.3-mo vs. 36.5-mo (HR: 0.66; 95% CI: 0.56–0.78; *P* < 0.001) rPFS: 33.0-mo vs. 14.8-mo (HR: 0.47; 95% CI: 0.39–0.55; *P* < 0.001)
STAPEDE	NCT00268476	Abiraterone vs. Placebo	Locally advanced prostate cancer or mHSPC	1917	3-yr OS rate: 83% vs. 76% (HR: 0.63; 95% CI: 0.52–0.76; *P* < 0.001) 3-yr FFS^b^ rate: 75% vs. 45% (HR: 0.29; 95% CI: 0.25–0.34; *P* < 0.001)
AFFIRM	NCT00974311	Enzalutamide vs. Placebo	mCRPC	1199	OS: 18.4-mo vs. 13.6-mo (HR: 0.63; 95% CI: 0.53–0.75; *P* < 0.001) rPFS: 8.3-mo vs. 2.9-mo (HR: 0.40; 95% CI: 0.35–0.47; *P* < 0.001)
PREVAIL	NCT01212991	Enzalutamide vs. Placebo	mCRPC	1717	OS: 32.4-mo vs. 30.2-mo (HR: 0.71; 95% CI: 0.60–0.84; *P* < 0.001) rPFS: NR vs. 3.9-mo (HR: 0.19; 95% CI: 0.15–0.23; *P* < 0.001)
ARCHES	NCT02677896	Enzalutamide vs. Placebo	mHSPC	1150	OS: NR vs. NR (HR: 0.66; 95% CI: 0.53–0.81; *P* < 0.001) rPFS: 49.8 vs. 38.9-mo (HR: 0.63; 95% CI: 0.52–0.76; *P* < 0.001)
ENZAMET	NCT02446405	Enzalutamide vs. First-generation antiandrogens^c^	mHSPC	1125	OS: NR vs. NR (HR: 0.70; 95% CI: 0.58–0.84; *P* < 0.001) cPFS^d^: 81.0 vs. 25.0-mo (HR: 0.45; 95% CI: 0.39–0.53; *P* < 0.001)
PROSPER	NCT02003924	Enzalutamide vs. Placebo	nmCRPC	1401	OS: 67.0-mo vs. 56.3-mo (HR: 0.73; 95% CI: 0.61–0.89; *P* = 0.001) MFS: 36.6 vs. 14.7-mo (HR: 0.29; 95% CI: 0.24–0.35; *P* < 0.001)
TITAN	NCT02489318	Apalutamide vs. Placebo	mHSPC	1050	OS: NR vs. 52.2-mo (HR: 0.65; 95% CI: 0.53–0.79; *P* < 0.001) CFS: NR vs. 11.4-mo (HR: 0.34; 95% CI: 0.29–0.41; *P* < 0.001)
SPARTAN	NCT01946204	Apalutamide vs. Placebo	nmCRPC	1207	MFS: 40.5-mo vs. 16.2-mo (HR: 0.28; 95% CI: 0.23–0.35; *P* < 0.001)
ACIS	NCT02257736	Apalutamide plus abiraterone vs. Abiraterone	mCRPC	982	rPFS: 24.0-mo vs. 16.6-mo (HR: 0.70; 95% CI: 0.60–0.83; *P* < 0.001)
ARAMIS	NCT02200614	Darolutamide vs. Placebo	nmCRPC	1509	MFS: 40.4-mo vs. 18.4-mo (HR: 0.41; 95% CI: 0.34–0.50; *P* < 0.001) 3-yr OS rate: 83% vs. 77% (HR: 0.69; 95% CI: 0.53–0.88; *P* = 0.003)
ARASENS	NCT02799602	Darolutamide plus docetaxel vs. Docetaxel	mHSPC	1306	OS: NR vs. 48.9-mo (HR: 0.68; 95% CI: 0.57–0.80; *P* < 0.001) CFS: NR vs. 19.1-mo (HR: 0.36; 95% CI: 0.30–0.42; *P* < 0.001)
ARANOTE	NCT04736199	Darolutamide vs. Placebo	mHSPC	669	rPFS: NR vs. 25-mo (HR: 0.54; 95% CI: 0.41–0.71; *P* < 0.001)
CHART	NCT03520478	Rezvilutamide vs. Bicalutamide	mHSPC	654	OS: NR vs. NR (HR: 0.58; 95% CI: 0.44–0.77; *P* < 0.001) rPFS: NR vs. 25.1-mo (HR: 0.44; 95% CI: 0.33–0.58; *P* < 0.001)

mCRPC: metastatic castration-resistant prostate cancer; mHSPC: metastatic hormone-sensitive prostate cancer; nmCRPC: non-metastatic castration-resistant prostate cancer; OS: overall survival; rPFS: radiographic progression-free survival; CFS: castration-free survival; FFS: failure-free survival; MFS: metastasis-free survival; cPFS: clinical progression-free survival; HR: hazard radio; CI: Confidence interval; NR: not reach.

a High-risk mHSPC: at least two of the three factors: Gleason score ≥8, more than three bone metastases, and visceral metastasis.

b FFS: PSA progression, death, or progression of local, lymph-node, or distant metastases.

c First-generation antiandrogens: bicalutamide, nilutamide, or flutamide.

d cPFS: radiographic progression, symptoms attributable to cancer progression, or initial of another anti-cancer treatment.

## 3 Mechanisms of resistance to NHT in prostate cancer

A comprehensive review of NHT applications in the treatment of advanced prostate cancer has been conducted (Supplementary material). While NHT demonstrates significant efficacy in delaying disease progression and enhancing patient survival, resistance to these therapies remains a formidable challenge. This section systematically examines resistance mechanisms from two primary perspectives: AR-dependent mechanisms and AR-independent mechanisms. Understanding these survival strategies employed by prostate cancer under NHT provides a theoretical foundation for developing novel strategies to address resistance in the future.

### 3.1 AR-dependent mechanisms

The therapeutic principle of NHT involves exerting their antitumor effects by inhibiting the AR pathway. However, prostate cancer cells can evade the effects of NHT through various AR-dependent mechanisms. This section examines these mechanisms, emphasizing their critical roles in resistance development.

#### 3.1.1 *AR* gene amplification

**Fig. 2:**
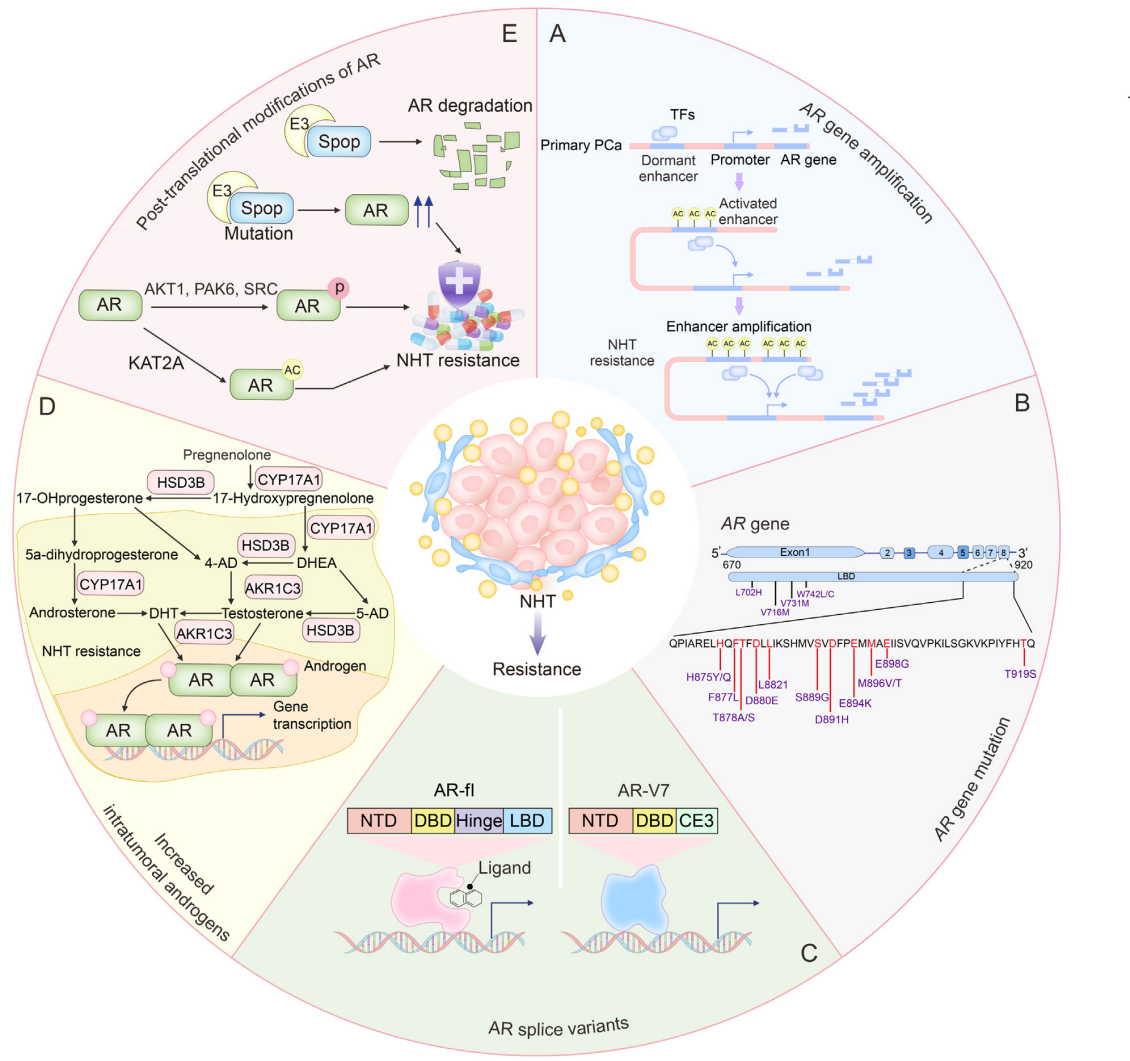
Androgen receptor (AR)-dependent mechanisms of resistance to novel hormone therapies (NHT) in prostate cancer. This figure illustrates the AR-dependent mechanisms that contribute to resistance against NHT in prostate cancer. The mechanisms are categorized into several sections: (A) *AR* gene amplification: Prostate cancer cells can amplify the *AR* gene, leading to increased AR signaling even under low androgen conditions, contributing to NHT resistance. (B) *AR* gene mutation: Specific mutations in the *AR* gene can lead to the activation of the AR pathway, bypassing the inhibitory effects of NHT drugs. (C) AR splice variants: Splice variants of AR, such as AR-V7, lack the ligand-binding domain and remain constitutively active, driving resistance to therapies targeting the AR pathway. (D) Increased intratumoral androgens: Prostate cancer cells can increase the synthesis of intratumoral androgens, activating the AR pathway despite systemic androgen deprivation. (E) Post-translational modifications of AR: Various post-translational modifications of the AR, including phosphorylation, acetylation, and ubiquitination, affect AR activity and stability, contributing to NHT resistance. Each section highlights a specific aspect of how prostate cancer cells adapt to and resist hormone therapies, illustrating the complexity and multifactorial nature of AR-dependent NHT resistance. PCa: prostate cancer; TF: transcription factor; AR-V7: androgen receptor splice variant 7; LBD: ligand-binding domain; NTD: N-terminal domain; DBD: DNA binding domain; CE3: cryptic exon 3; HSD3B: 3β-hydroxysteroid dehydrogenase; AKR1C3: aldo-keto reductase family 1 member C3; CYP17A1: cytochrome P450 17A1; AKT1: protein kinase B 1; PAK6: p21 (RAC1) activated kinase 6; SRC: SRC proto-oncogene, non-receptor tyrosine kinase; KAT2A: lysine acetyltransferase 2A.

*AR* gene amplification is a central mechanism by which prostate cancer develops resistance to NHT (Fig. 2A). Increased *AR* gene copy number allows cancer cells to sustain active AR signaling even under reduced androgen levels. Over 50% of castration-resistant prostate cancer (CRPC) patients exhibit *AR* gene amplification [5]. Although the origins of this phenomenon are not fully understood, genetic instability and selective evolutionary pressures are thought to be contributing factors. Recent whole-genome sequencing studies indicate that a potent enhancer located approximately 650–700 kb upstream of the *AR* gene may drive this amplification [6].

#### 3.1.2 *AR* gene mutation

Specific mutations in the *AR* gene represent another cause of resistance to NHT (Fig. 2B). For example, treatment failures with abiraterone, a CYP17A1 enzyme inhibitor, have been linked to the accumulation of progesterone. This byproduct activates mutant *AR* genes, such as *AR* T878R and *AR* H875Y, enabling the AR pathway to bypass abiraterone's inhibitory effects [7]. Moreover, certain *AR* mutants, including *AR* F876L, can convert enzalutamide's inhibitory effects, thereby inducing resistance to enzalutamide [8].

#### 3.1.3 AR splice variants

The ligand-binding domain (LBD) is a crucial structure within the AR protein, responsible for recognizing and binding androgens. AR splice variants lacking the LBD but retaining the N-terminal domain (NTD) and DNA-binding domain (DBD) remain constitutively active in the absence of androgen binding, thereby activating downstream signaling pathways and driving prostate cancer resistance and progression (Fig. 2C) [9]. Among these variants, AR splice variant 7 (AR*-*V7*)* has garnered the most attention and has been extensively studied [10]. Elevated levels of AR-V7 protein are strongly associated with resistance to abiraterone and enzalutamide. Knocking down AR*-*V7 or using AR-V7 antagonists can restore drug sensitivity in resistant cells, while overexpressing AR-V7 in sensitive cells can induce resistance [11]. Clinical studies have also shown that detecting AR-V7 in circulating tumor cells predicts poor outcomes for mCRPC patients treated with abiraterone and enzalutamide [12].

#### 3.1.4 Increased intratumoral androgens

Increased synthesis of intratumoral androgens significantly contributes to resistance against NHT in prostate cancer (Fig. 2D) [13]. Mutations or overexpression of enzymes in androgen metabolic pathways enable prostate cancer cells to synthesize androgens independently, thus bypassing the need for exogenous androgens and activating the AR pathway, which leads to resistance. Notably, enzymes such as 3β-hydroxysteroid dehydrogenase type 1 (HSD3B1) and aldo-keto reductase family 1 member C3 (AKR1C3), play pivotal roles in this process. The N367T mutant of HSD3B1 increases the enzyme's stability and activity, enabling CRPC cells to convert residual dehydroepiandrosterone into active androgens more easily, thus leading to resistance [14]. Studies have demonstrated that mutations in HSD3B1 can convert abiraterone from an androgen synthesis inhibitor to an AR agonist, thereby further enhancing AR signaling activity [15]. AKR1C3, a member of the aldo-keto reductase superfamily, plays a critical role in developing resistance to AR-targeted therapies. Weaker AR-binding androgens, including androstenedione and 5-alpha-androstenedione, are converted into testosterone and dihydrotestosterone, which have a higher AR activation potential, through catalysis by AKR1C3 [16]. Studies have shown that AKR1C3 enhances the AR pathway activity by promoting endogenous androgen synthesis, ultimately leading to CRPC cell resistance to abiraterone and enzalutamide. Moreover, downregulating AKR1C3 or inhibiting its enzymatic activity can restore drug sensitivity to abiraterone and enzalutamide in resistant CRPC cells [17,18]. Furthermore, research has demonstrated that AKR1C3 enhances the protein stability of AR-V7 through the ubiquitination pathway and synergizes with AR-V7 to promote resistance to anti-androgen therapy in CRPC cells [19]. Furthermore, a clinical study involving 163 mCRPC patients verified that AKR1C3 expression is associated with early resistance to abiraterone, with AKR1C3-positive patients exhibiting significantly shorter prostate-specific antigen progression-free survival (PSA-PFS) and radiographic progression-free survival (rPFS) compared to AKR1C3-negative patients (median PSA-PFS: 5.7-mo vs. 11.2-mo, *P* < 0.001; median rPFS: 12.4-mo vs. 23.3-mo, *P* = 0.048) [20].

#### 3.1.5 Post-translational modifications of AR

Post-translational modifications of the AR protein, including phosphorylation, acetylation, and ubiquitination, significantly impact its activity and stability. Abnormal modification processes contribute to resistance against NHT (Fig. 2E). For instance, AR phosphorylation, influenced by various protein kinases, can result in abnormal phosphorylation due to the abnormal activity of these kinases, potentially making the AR overly sensitive to low concentrations of androgens and thus promoting resistance in prostate cancer cells [21]. Acetylation of the AR can affect its activation, nuclear translocation, and transcriptional activity, potentially impacting resistance to prostate cancer treatments. Studies have demonstrated that lysine acetyltransferase 2A (KAT2A) can promote CRPC resistance to abiraterone by acetylating the AR [22]. AR ubiquitination, a primary pathway for AR protein degradation, can also contribute to resistance if modified. For example, mutations in the speckle-type POZ protein (SPOP), a substrate recognition component of the E3 ubiquitin ligase, can disrupt normal AR ubiquitination, leading to increased AR protein stability and enhanced AR signaling, ultimately increasing resistance in prostate cancer cells [23].

### 3.2 AR-independent pathways

Beyond the AR-dependent mechanisms previously discussed, AR-independent pathways also contribute to NHT resistance.

#### 3.2.1 Glucocorticoid receptor (GR)/progesterone receptor (PR) pathway

**Fig. 3:**
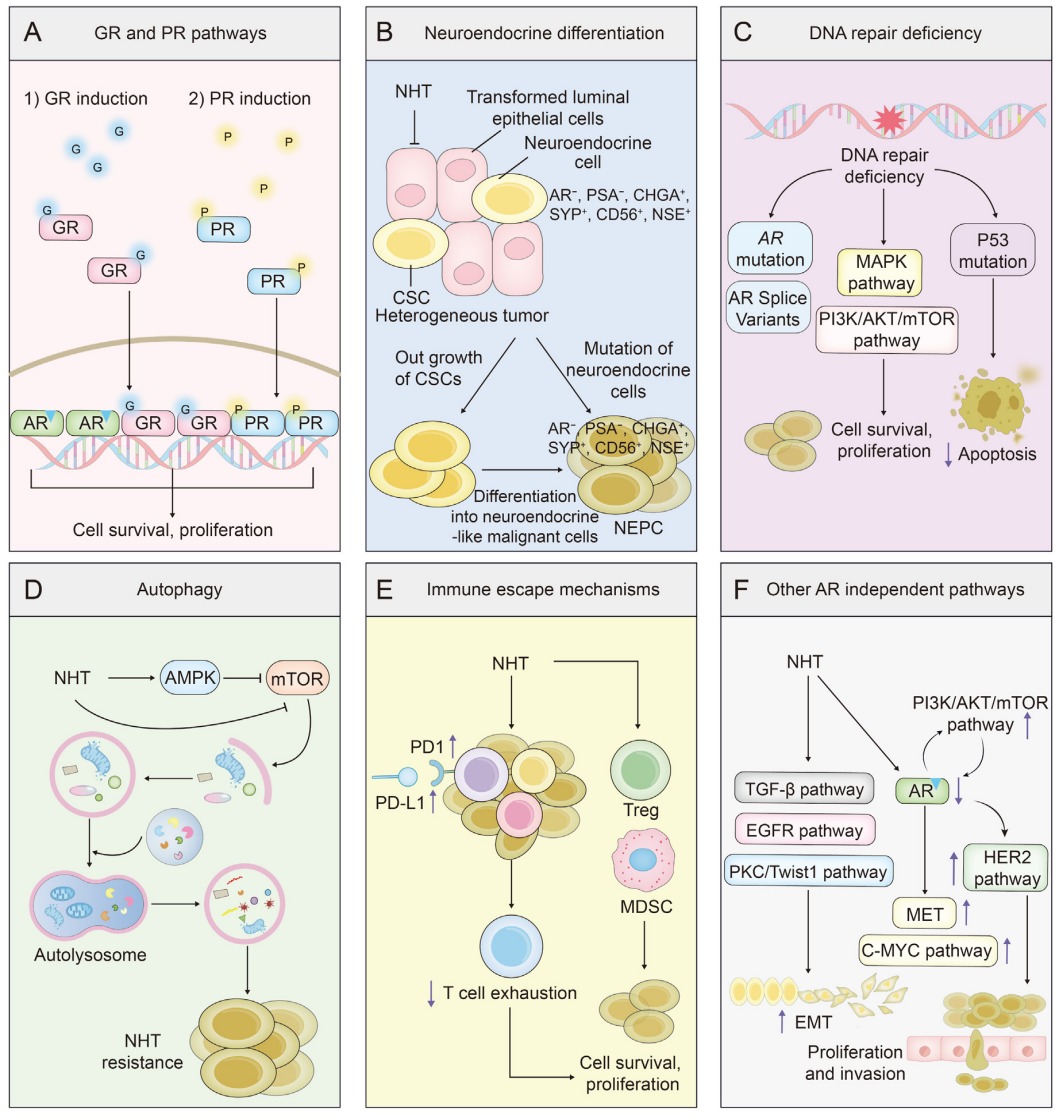
Androgen receptor (AR)-independent mechanisms of resistance to novel hormone therapies (NHT) in prostate cancer. This figure illustrates the AR-independent mechanisms that contribute to resistance against NHT in prostate cancer. The mechanisms are categorized into several sections: (A) GR and PR Pathways: GR and PR pathways can be upregulated under endocrine therapy pressure, sustaining cell survival and proliferation through shared signaling pathways with AR. (B) Neuroendocrine differentiation: prostate cancer cells can undergo neuroendocrine differentiation, resulting in a cell phenotype that is less dependent on AR signaling. This includes the emergence of NEPC cells. (C) DNA repair deficiency: deficiencies in DNA repair mechanisms, such as mutations in DNA repair genes, can lead to genomic instability and resistance to endocrine therapies. This includes the activation of compensatory signaling pathways like PI3K/AKT/mTOR and MAPK pathways. *AR* mutations, including the production of AR splice variants like AR-V7, and mutations in tumor suppressor genes such as *TP53* also contribute significantly to therapy resistance by preventing apoptosis and promoting cell survival. (D) Autophagy: endocrine therapies can induce autophagy, a process that helps cancer cells survive by degrading and recycling cellular components. (E) Immune escape mechanisms: endocrine therapies can lead to immune evasion by upregulating immune checkpoint proteins such as PD-1/PD-L1, leading to T cell exhaustion and reduced anti-tumor immunity. Increased proportions of Treg and MDSC in the tumor microenvironment also contribute to immune evasion. (F) Other AR-independent pathways: additional pathways contribute to resistance, with TGF-β, EGFR, and PKC/Twist1 promoting EMT, and HER2, MET, C-MYC, and PI3K/AKT/mTOR promoting cell proliferation and invasion independently of AR signaling. Each section highlights how prostate cancer cells can bypass AR dependency and develop resistance to hormone therapies through various alternative mechanisms. GR: glucocorticoid receptor; PR: progesterone receptor; NEPC: neuroendocrine prostate cancer; PSA: prostate-specific antigen; CHGA: chromogranin A; SYP: synaptophysin; CD56: neural cell adhesion molecule 1; NSE: neuron-specific enolase; CSC: cancer steam cell; PI3K: phosphoinositide 3-kinase; AKT: protein kinase B; mTOR: mechanistic target of rapamycin; MAPK: mitogen-activated protein kinase; TP53: tumor protein p53; AMPK: AMP-activated protein kinase; PD-1: programmed cell death protein 1; PD-L1: programmed death-ligand 1; Treg: regulatory T cells; MDSC: myeloid-derived suppressor cells; TGF-β: transforming growth factor-beta; EGFR: epidermal growth factor receptor; PKC: protein kinase C; HER2: human epidermal growth factor receptor 2; MET: mesenchymal-epithelial transition factor; C-MYC: myelocytomatosis oncogene; EMT: epithelial-mesenchymal transition.

As shown in Fig. 3A, the GR and PR influence resistance to NHT in prostate cancer. This occurs because *GR* and *PR* share numerous signaling pathways with AR. Under the therapeutic pressure of NHT, prostate cancer cells may upregulate GR and PR, activating pathways typically stimulated by AR, thereby sustaining tumor cell proliferation and survival [24,25].

#### 3.2.2 Neuroendocrine prostate cancer (NEPC)

NEPC represents another form of resistance to NHT in prostate cancer (Fig. 3B). Current research indicates that aside from a few cases of untreated NEPC, most NEPC develops in late-stage prostate cancer patients who have undergone multiple rounds of anti-androgen therapy. The prevailing view posits that neuroendocrine differentiation in prostate cancer is an adaptive response to a low-androgen environment after prolonged androgen suppression therapy, although the specific mechanisms remain unclear [26]. NEPC typically has low or absent AR expression and exhibits neuroendocrine markers, including chromogranin A (CgA) and neuron-specific enolase (NSE) [27]. Additionally, NEPC is frequently associated with mutations in tumor suppressor genes such as retinoblastoma 1 (RB1), tumor protein p53 (TP53), and phosphatase and tensin homolog (PTEN), as well as overexpression of the potent transcription factor MYCN and abnormal activation of growth-promoting signaling pathways such as phosphoinositide 3-kinase (PI3K)/protein kinase B (AKT) [28,29]. These factors could promote tumor proliferation and survival independently of the AR pathway.

#### 3.2.3 DNA repair defects

DNA repair defects represent another crucial mechanism contributing to resistance to NHT (Fig. 3C). These defects prevent prostate cancer cells from effectively repairing DNA damage, resulting in increased genomic instability. Consequently, cancer cells are more likely to acquire mutations under therapeutic pressure, including those that facilitate escape from endocrine therapy [30,31]. For instance, cancer cells with DNA repair defects may acquire *TP53* mutations, rendering them more resistant to DNA damage and enabling them to evade apoptosis [32,33]. Moreover, DNA repair defects increase the incidence of *AR* gene mutations and AR splice variants [34]. On the other hand, these defects can prompt prostate cancer cells to activate compensatory signaling pathways, such as the PI3K/AKT/mechanistic target of rapamycin (mTOR) or mitogen-activated protein kinase (MAPK) pathways, to sustain cell survival and progression [35].

#### 3.2.4 Autophagy

Autophagy is an intracellular degradation and recycling mechanism that plays a pivotal role in responding to cellular stress and maintaining cellular homeostasis [36]. As shown in Fig. 3D, enzalutamide can induce autophagy by activating AMP-activated protein kinase (AMPK) and inhibiting the mTOR pathway; autophagy aids prostate cancer cells in surviving endocrine therapy pressure by meeting metabolic demands and degrading intracellular anticancer drugs or related proteins, thus reducing drug efficacy and promoting resistance [[37], [38], [39]].

#### 3.2.5 Immune evasion

Although prostate cancer is typically considered an immune-cold tumor, immune evasion mechanisms can still influence endocrine therapy resistance in specific cases (Fig. 3E) [40,41]. Studies show that endocrine therapy increases the expression of programmed cell death protein 1 (PD-1) and programmed cell death ligand 1 (PD-L1) in tumor cells and the tumor microenvironment. The activation of the PD-1/PD-L1 pathway results in T cell exhaustion, diminishing the ability of the immune system to attack tumor cells, thereby facilitating prostate cancer cell growth and dissemination under endocrine therapy [42]. Additionally, endocrine therapy can elevate the proportion of immunosuppressive cells in the prostate cancer tumor microenvironment (TME), including regulatory T cells and myeloid-derived suppressor cells, further dampening local antitumor immune responses [40].

#### 3.2.6 Other AR-independent pathways

Other AR-independent pathways related to NHT resistance are summarized in Fig. 3F. Epithelial-mesenchymal transition (EMT) represents the process through which cells transit from an epithelial to a mesenchymal phenotype [43], endowing them with enhanced invasiveness and migratory capabilities. Research suggests that endocrine therapy can induce EMT via diverse signaling pathways, including the transforming growth factor beta (TGF-β), protein kinase C (PKC)/twist family BHLH transcription factor 1 (Twist1), and epidermal growth factor receptor (EGFR) signaling pathways [[44], [45], [46]]. Moreover, the PI3K/AKT/mTOR signaling pathway plays a pivotal role in cell cycle regulation, growth, proliferation, and apoptosis [47], with its aberrant activation markedly contributing to endocrine therapy resistance in prostate cancer [48]. Studies have demonstrated a reciprocal activation relationship between the AR and PI3K pathways, whereby inhibition of AR signaling leads to activation of the PI3K pathway, and vice versa [49]. Human epidermal growth factor receptor 2 (HER2), a tyrosine kinase receptor, is closely associated with the progression and treatment resistance of various cancers, including prostate cancer [50]. Research demonstrates that inhibition of the AR pathway by enzalutamide can upregulate the HER2 signaling pathway, thereby enabling prostate cancer cells to sustain growth and survival through this compensatory mechanism [51]. Similar compensatory mechanisms are noted in the hepatocyte growth factor (HGF)/mesenchymal-epithelial transition factor (MET) and myelocytomatosis oncogene (C-MYC) signaling pathways. The HGF/MET and C-MYC pathways play critical roles in cancer proliferation, invasion, and treatment resistance [52,53]. In prostate cancer, overexpression of MET and C-MYC is associated with more aggressive cancer phenotypes [54,55]. The AR pathway negatively regulates the expression of MET and C-MYC; thus, when endocrine therapy reduces androgen levels and inhibits the AR pathway, the inhibitory effect on MET and C-MYC is lessened, leading to the upregulation of MET and C-MYC signaling pathways, which ultimately results in increased cancer cell growth, dissemination and resistance [[56], [57], [58]].

## 4 Strategies for countering resistance to NHT

### 4.1 Sequential treatment with novel AR-targeted drugs

Due to structural and mechanistic similarities, cross-resistance exists among NHT. However, most studies on sequential therapy have focused on earlier drugs such as abiraterone and enzalutamide, demonstrating limited survival benefits for CRPC patients undergoing sequential therapy with these agents [59]. Some studies suggest that sequencing abiraterone followed by enzalutamide may offer more benefits in improving disease progression-free survival than the reverse sequence, although no significant differences in overall survival (OS) have been noted [60]. Apalutamide and darolutamide, as newer drugs with limited cross-resistance studies, have shown potential in improving outcomes for patients previously treated with abiraterone and enzalutamide. Studies indicate that apalutamide could extend PSA progression-free survival by an additional 3.7 months for such patients [61]. Additionally, since mutations like *AR* T877A, *AR* W742L, and *AR* F876L are critical in resistance to flutamide, bicalutamide, enzalutamide and apalutamide, darolutamide's ability to inhibit these mutants suggests its potential to enhance effectiveness against AR-targeted drugs. Preclinical research has confirmed that darolutamide can inhibit the proliferation of enzalutamide-resistant cells [8]. Overall, emerging AR-targeted drugs may hold potential in addressing resistance, yet more robust clinical evidence is required to substantiate their effectiveness and determine their optimal use in prostate cancer therapy.

### 4.2 Docetaxel

Docetaxel exerts its antitumor effects by stabilizing microtubules and inhibiting their depolymerization, thus preventing cell mitosis. Docetaxel represents another essential therapeutic approach for CRPC and is traditionally recommended as a first-line treatment option. However, with the emergence of more convenient and less toxic NHT, physicians and patients are increasingly opting for NHT over docetaxel as a first-line treatment. For patients who have not previously undergone chemotherapy but have developed resistance to NHT, docetaxel is recommended as a second-line treatment. It is crucial to note that docetaxel, used as a subsequent treatment following resistance to NHT, exhibits cross-resistance. Specifically, preclinical studies have demonstrated that abnormal activation of the AR is associated not only with resistance to AR*-*targeted drugs but also with docetaxel, whose target—cellular microtubules—could interact with the AR, revealing cross-resistance to taxanes in cells resistant to enzalutamide [62,63]. Clinical studies have also indicated that the detection of continuous AR nuclear translocation in circulating tumor cells predicts poor outcomes with docetaxel [64]. The combination of docetaxel and novel AR-targeted drugs may be a potential treatment. As previously described, the Phase III RCT ARASENS demonstrated that the combination of docetaxel and darolutamide provides a therapeutic advantage over docetaxel alone in patients with mHSPC [7]. In a preclinical study based on organoids and primary cells, the combination of docetaxel and darolutamide exhibited significant antitumor effects in CRPC model that had developed resistance to docetaxel, enzalutamide, and cabazitaxel [65]. Additionally, a Phase II clinical trial is currently assessing the efficacy and safety of rezvilutamide combined with docetaxel in chemotherapy-naive mCRPC patients who have progressed following abiraterone treatment. According to recent conference reports, the PSA response rate at 12 weeks was 67.7%, with 75.0% of patients achieving a PSA reduction of ≥50% [66].

### 4.3 Poly ADP-ribose polymerase inhibitors (PARPI)

DNA single-strand break repair and double-strand break repair constitute dual mechanisms that ensure accurate DNA replication in response to DNA damage. Some tumors with mutations in homologous recombination repair (HRR) genes are unable to effectively perform double-strand break repair and primarily depend on single-strand break repair for DNA repair. Single-strand break repair relies on poly ADP-ribose polymerase (PARP); thus, PARPI inhibit PARP activity, preventing single-strand break repair and ultimately inducing "synthetic lethality" to eliminate tumor cells with HRR gene mutations.

Several clinical trials have confirmed that PARPI significantly enhance the prognosis of HRR-deficient patients who progress following NHT [67]. Preclinical studies also indicate no cross-resistance between NHT drugs and PARPI [68]. However, PARPI are only approved for mCRPC patients with specific DNA damage repair gene mutations, including breast cancer susceptibility gene 1/2 (*BRCA1/2*) and ataxia telangiectasia mutated (*ATM*), thus many patients resistant to NHT drugs do not benefit from PARPI. Consequently, ongoing research is crucially focused on identifying more patients who could benefit from PARPI. In fact, studies suggest that the homologous recombination deficiency (HRD) score, which integrates three DNA-based measures of genomic instability, may assist in identifying more prostate cancer patients who could benefit from PARPI [69].

Combining PARPI with NHT represents another strategy to overcome resistance and delay disease progression. Basic research indicates that the AR pathway interacts with DNA repair genes, with this pathway regulating the expression of several such genes; endocrine therapy can induce a "BRCAness" phenotype in cancer cells, thereby increasing their sensitivity to PARPI [34,70]. In clinical research, the results of Phase III RCTs such as PROpel, MAGNITUDE, and TALAPRO-2 have demonstrated promising outcomes for the combined use of PARPI and NHT [[71], [72], [73]]. In the PROpel trial, the combination of olaparib and abiraterone significantly prolonged rPFS and OS in HRR-deficient patients compared to abiraterone alone. For HRR-proficient patients, the combination therapy significantly prolonged rPFS compared to abiraterone alone, yet no difference in OS was observed between the two groups. Preliminary results from the MAGNITUDE trial indicated that the combination of Niraparib with abiraterone significantly prolonged rPFS in HRR-deficient patients, particularly those with *BRCA* mutations, compared to abiraterone alone, though no significant benefit was observed in HRR-proficient patients. In the TALAPRO-2 trial, the combination of talazoparib and enzalutamide significantly extended rPFS in HRR-deficient patients, especially those with *BRCA* mutations, compared to enzalutamide alone. Furthermore, the combination therapy also significantly improved rPFS in HRR-proficient or HRR status-unknown patients. Based on the current results of these three clinical trials, the combination of PARPI and NHT shows definite efficacy in HRR-deficient patients. However, for HRR-proficient patients, this combined strategy does not improve survival. It is noteworthy that nearly all patients included in these studies had not previously received NHT treatment. The efficacy of the PARPI and NHT combination strategy in patients who have developed resistance to NHT is yet to be determined and requires further investigation in future studies.

### 4.4 Radionuclide therapy

Radionuclide therapy represents another type of post-NHT failure treatment. Ra223, a radioactive drug, emits alpha particles, inducing double-strand DNA breaks in tumor cells resulting in potent tumoricidal effects. In 2013, Ra223 received US Food and Drug Administration (FDA) approval for treating mCRPC patients with symptomatic bone metastases and no visceral metastases, based on its significant extension of OS and reduction of skeletal-related events demonstrated in the Phase III ALSYMPCA study [74]. Current studies are actively exploring the efficacy of combination therapy strategies involving Ra223. The combination of Ra223 and abiraterone was discouraged following negative results from the Phase III ERA223 study, which indicated no improvement in survival and an increased incidence of fractures [75]. A Phase II clinical trial demonstrated that the combination of Ra223 and enzalutamide extended PFS compared to enzalutamide alone in mCRPC patients, prompting the ongoing Phase III PEACE III study (NCT02194842) to further evaluate this combination strategy's efficacy [76]. Another recent Phase I clinical trial (NCT03737370) documented the efficacy and safety of Ra223 combined with docetaxel in CRPC patients who failed NHT, revealing a PSA-PFS of 11.7 months and a PSA50 response rate of 51.4% [77]. Additionally, preliminary exploration of the combination of Ra223 and PARPI has been conducted in clinical trials. Phase I clinical trials (NCT03317392 and NCT03076203) have established the safety and preliminary clinical efficacy of Ra223 combined with Niraparib and Olaparib in mCRPC patients who failed multiple lines of therapy, including NHT [78,79].

Lu177-PSMA-617 represents another emerging effective treatment strategy for NHT-resistant cases. PSMA is highly expressed in prostate cancer and minimally in normal tissues, identifying it as a crucial target for prostate cancer imaging and treatment. Lu177-PSMA-617, a radioligand therapy, delivers beta radiation to PSMA-expressing prostate cancer cells and received FDA approval in 2022 for mCRPC. The approval of Lu177-PSMA-617 primarily rested on improved rPFS and OS demonstrated in the VISION Phase III RCT [80]. Notably, the VISION trial included 831 mCRPC patients who had received at least one NHT and progressed, highlighting Lu177's effectiveness as a post-NHT failure treatment strategy. Recent results from the Phase III PSMAfore trial, which enrolled 585 PSMA-positive, taxane-naive patients with mCRPC, demonstrate that Lu177-PSMA-617 not only prolongs rPFS but also results in fewer adverse events (AEs) compared to switching to an unused alternative NHT, either abiraterone or enzalutamide [81]. These findings suggest that Lu177-PSMA-617 offers a more effective and safer therapeutic alternative for PSMA-positive mCRPC patients after NHT failure. Numerous ongoing studies are assessing whether the combination of Lu177-PSMA-617 with other treatments can enhance the depth and durability of treatment responses. Clinical trials are actively exploring the combinations of Lu177 with NHT (NCT04419402 and NCT04720157), PARPI (NCT03874884 and NCT03317392), chemotherapy (NCT05340374 and NCT04343885), and immunotherapy (NCT03658447 and NCT05150236), with results that warrant close attention. Overall, radionuclide therapy and its combination strategies will be crucial areas of exploration to delay disease progression after NHT failure.

### 4.5 Bipolar androgen therapy

Prostate cancer cells can adapt to low androgen environments by increasing AR levels, which leads to resistance. Research has shown that high doses of androgens can suppress or kill cells with elevated AR expression. Consequently, bipolar androgen therapy (BAT) has been developed as an innovative treatment strategy based on this finding. BAT involves administering high doses of testosterone periodically to create a 'peak and valley' cycle of androgen levels in the body, ultimately achieving antitumor effects by killing resistant cells [82]. The TRANSFORMER clinical trial, enrolling 195 mCRPC patients progressing under abiraterone treatment, confirmed the efficacy and safety of BAT. The trial revealed that 28.2% of patients experienced more than a 50% reduction in PSA levels, and BAT also prolonged the effective period of subsequent enzalutamide treatment [83]. However, further exploration in larger clinical trials is still required to determine the long-term effects and optimal application of BAT.

### 4.6 Strategies targeting endogenous androgen synthesis and AR splice variants

Given AKR1C3's pivotal role in endogenous androgen synthesis and resistance mechanisms to AR-targeted drugs, AKR1C3 inhibitors have attracted significant attention for their potential to counteract resistance. Research has shown that indomethacin, an AKR1C3 inhibitor, can restore sensitivity to abiraterone and enzalutamide in resistant cells [17,18]. A Phase I/II trial (NCT02935205) explored the efficacy and safety of combining indomethacin and enzalutamide in mCRPC patients, but the results have not yet been disclosed.

The crucial role of AR splice variants in resistance mechanisms has sparked interest in antagonists specifically targeting these variants. Studies have suggested that niclosamide, an AR-V7 antagonist, can restore drug sensitivity in resistant cells [11,84]. Several ongoing Phase I or II clinical trials (NCT03123978, NCT02532114, and NCT028077805) are investigating the efficacy and safety of combining niclosamide with enzalutamide or abiraterone, and the results of these trials are eagerly awaited. Additionally, EPI-001, a small molecule antagonist that covalently binds to the AR NTD, has been shown to decrease the transcriptional activity of AR and its splice variants, with animal studies confirming its effectiveness in inhibiting CRPC xenograft growth [85]. Heat shock protein 90 (HSP90) is crucial for the maturation, activation, and stabilization of AR. Experimental evidence has shown that bruceantin, an HSP90 inhibitor, disrupts the interaction between HSP90 and AR/AR-V7, leading to the ubiquitination and degradation of AR/AR-V7 and effectively inhibiting the growth and metastasis of CRPC xenografts in mice [86]. A Phase I/II clinical trial (NCT01685268) is currently exploring the efficacy and safety of combining the HSP90 inhibitor AT13387 with abiraterone in CRPC patients, but the results have not yet been disclosed.

### 4.7 Strategies targeting the degradation of AR

In recent years, AR degraders have emerged as a promising therapeutic strategy for the treatment of CRPC. These degraders differ from traditional AR antagonists in that they overcome resistance by promoting AR protein degradation. Among these, proteolysis-targeting chimeras (PROTAC)-based AR degraders have demonstrated significant breakthroughs. These compounds function by recruiting E3 ubiquitin ligases, which tag AR proteins for degradation via the ubiquitin-proteasome system [87].

PROTAC-based AR degraders, including ARV-110 (NCT03888612 and NCT05177042), ARV-766 (NCT05067140), CC-94676 (NCT04428788), HP-518 (NCT05252364), AC176 (NCT05241613), and GT-20029 (NCT05428449), have entered early-stage clinical trials [88]. Currently, only ARV-110 and ARV-766 have progressed to Phase II clinical trials. In clinical trials, ARV-110 and ARV-766 have shown significant antitumor efficacy, along with favorable tolerability and safety, in prostate cancer patients who had progressed following multiple lines of therapy, including at least one NHT [89,90]. ARV-110 has notably exhibited an impressive ability to degrade mutated AR (T878X and H875Y) [91]. ARV-766, an optimized PROTAC derived from ARV-110, is capable of effectively degrading a broader spectrum of AR mutants, including T878A, H875Y, and L702H etc. [90]. Additionally, it is important to highlight that CC-94676 is a dual-function drug, combining AR antagonism with AR degradation. The most recent Phase I clinical trial, which included 95 patients with mCRPC who had undergone multiple lines of therapy (including at least one NHT), indicated that CC-94676 conferred benefits in reducing PSA levels and prolonging rPFS, while exhibiting favorable tolerability and safety [92].

The progression of these studies suggests that AR degraders hold significant potential for the treatment of CRPC, particularly in patients resistant to NHT. However, despite the encouraging results observed in early clinical trials, their efficacy and safety in broader clinical practice remain to be validated. In conclusion, research on AR degraders remains in its developmental stage, and continuous attention to emerging advancements in this field is imperative.

### 4.8 Strategies targeting AR-independent pathways

As previously mentioned, AR-independent pathways play a crucial role in contributing to resistance to endocrine therapy (Fig. 3). Several treatment strategies targeting AR-independent pathways have demonstrated potential in overcoming endocrine therapy resistance.

Preclinical studies have shown that targeting the GR/PR can restore cellular sensitivity to AR-targeted drugs [93]. However, current clinical trials have yet to provide evidence supporting this strategy. A Phase I/II clinical trial (NCT02012296) investigated the efficacy and safety of combining enzalutamide with the GR/PR antagonist mifepristone in treating mCRPC. The results revealed that adding mifepristone did not significantly extend PSA-PFS or rPFS compared to enzalutamide alone [94]. Further exploration of more suitable GR/PR antagonists is needed in future research.

NEPC represents a rare and highly aggressive form of prostate cancer with poor clinical outcomes. Treatment for NEPC primarily draws from strategies used for tumors with neuroendocrine characteristics, such as small cell lung cancer (SCLC). Currently, platinum-based chemotherapy, taxane-based chemotherapy, and etoposide are the primary treatment options for NEPC. Platinum-based chemotherapy (e.g., cisplatin and carboplatin) functions by forming cross-links with DNA, leading to double-strand breaks that inhibit DNA replication and transcription, ultimately resulting in NEPC cell death. Etoposide, a topoisomerase II inhibitor, prevents DNA replication and transcription by inhibiting topoisomerase II activity, thereby resulting in NEPC cell death [95]. Etoposide is frequently combined with cisplatin or carboplatin for NEPC treatment due to NEPC's typical DNA repair deficiencies, which render them highly sensitive to platinum-based drugs and etoposide [96]. Taxane-based drugs (e.g., docetaxel, paclitaxel, and cabazitaxel) inhibit microtubule depolymerization, prevent cell mitosis, induce apoptosis, and inhibit angiogenesis, thereby exerting antitumor effects on NEPC. Despite the effectiveness of these existing therapies for NEPC, resistance continues to pose a significant challenge. Research indicates that cancer cells can develop resistance to platinum-based drugs and etoposide through increased drug efflux, enhanced DNA damage repair, and modulation of topoisomerase II expression [97,98]; similarly, resistance to taxane-based drugs can emerge through alterations in microtubule protein structure or expression of resistance genes [99]. Several emerging therapies for NEPC, including PARPI, immune checkpoint inhibitors, and tyrosine kinase inhibitors, are currently undergoing preclinical or early clinical trials. However, their efficacy in treating NEPC has not yet been established in Phase III RCTs [100].

Although immune evasion mechanisms following endocrine therapy may represent one of the AR-independent pathways leading to endocrine therapy resistance, the application of immunotherapy in prostate cancer remains limited. To date, only two immunotherapy approaches have received FDA approval for mCRPC. Firstly, pembrolizumab is approved for a very small subset of CRPC patients with microsatellite instability-high (MSI-high) or mismatch repair-deficient (dMMR) genetic features who have failed standard treatments. Secondly, sipuleucel-T is designed to gradually enhance the immune response against cancer cells. However, sipuleucel-T is only suitable for asymptomatic or minimally symptomatic mCRPC and is not ideal for rapidly progressing mCRPC following NHT failure. Moreover, due to its complex treatment process and high cost, sipuleucel-T has not been widely adopted in clinical practice. Despite the limited application of immunotherapy in advanced prostate cancer, numerous clinical trials are investigating its efficacy in late-stage prostate cancer after NHT failure. Early small-sample single-arm clinical trials, like the Phase Ib study KEYNOTE-028 and the Phase II study KEYNOTE-199, reported preliminary antitumor activity and disease control rates of pembrolizumab in mCRPC patients who had failed standard treatments, including NHT [101,102]. However, disappointingly, recent large-sample Phase III RCTs—KEYNOTE-641, KEYLYNK-010, and KEYNOTE-921—announced that pembrolizumab combined with enzalutamide, PARPI, and docetaxel, respectively, failed to significantly improve the prognosis of mCRPC patients who had failed standard treatments, primarily NHT [[103], [104], [105]]. The Phase II study CheckMate-9KD assessed the efficacy and safety of another PD-1 antibody, nivolumab, in combination with various treatments in mCRPC patients who had progressed after NHT. The results indicated that combinations of nivolumab with rucaparib, docetaxel, or enzalutamide demonstrated efficacy and acceptable safety profiles; however, further research in Phase III clinical trials is needed to confirm if the addition of nivolumab offers a survival benefit over monotherapy strategies [106,107]. The Phase III RCT IMbassador250 showed that for CRPC patients who failed abiraterone treatment, combining atezolizumab with enzalutamide did not significantly improve OS or rPFS compared to enzalutamide alone, and PD-L1 expression was ineffective in identifying patients who would benefit from the combination therapy [108]. Regarding ipilimumab, previous clinical trials (NCT01057810) have demonstrated that ipilimumab alone does not offer a benefit for CRPC patients who have failed endocrine therapy. However, the Phase II study CheckMate650 found that combining ipilimumab with nivolumab has potential therapeutic efficacy, especially for prostate cancer patients with a high tumor mutational burden. Nevertheless, the feasibility of this combination strategy still requires further validation in future Phase III clinical trials [109].

PKC inhibitors and metformin have been shown to reverse enzalutamide resistance in prostate cancer cells by inhibiting EMT [44,110]. Additionally, metformin has also been demonstrated to restore drug sensitivity in enzalutamide-resistant cells by inhibiting autophagy [38]. Currently, ongoing clinical trials (NCT02640534 and NCT02339168) are exploring the efficacy and safety of combining metformin with enzalutamide, and the results of these trials are eagerly awaited.

Various PI3K/AKT/mTOR pathway inhibitors have shown potential in preclinical models to reverse resistance when combined with endocrine therapy [111,112]. In clinical trials, the Phase III RCT IPATential150 confirmed that in treatment-naive mCRPC patients with PTEN loss, the AKT inhibitor ipatasertib combined with abiraterone significantly extended rPFS compared to abiraterone alone [113]. Another Phase Ib clinical trial demonstrated that the combination of the mTOR inhibitor CC-115 and enzalutamide had good efficacy and safety in treatment-naive mCRPC patients [114]. For patients who have progressed after NHT, an Ib/II clinical trial explored the efficacy and safety of the PI3K/mTOR inhibitor samotolisib combined with enzalutamide in mCRPC patients who had progressed after abiraterone. The results showed that the combination therapy significantly extended PFS and rPFS compared to enzalutamide alone, especially in patients with intact PTEN and AR-V7 negative status [115]. Finally, preclinical studies have shown that inhibitors of the C-MYC, HER2, and MET pathways also have potential to improve resistance to endocrine therapy; however, clinical trial evidence is currently lacking [[116], [117], [118]].

## 5 Discussion and looking forward

NHT have had a profound impact on the treatment of advanced prostate cancer, significantly enhancing patient survival and quality of life. Initially, NHT was primarily used in CRPC, which is the terminal stage of the disease. However, as NHT has become more widely used in HSPC, the issue of NHT resistance has emerged as an unavoidable challenge in the comprehensive management of patients with advanced prostate cancer. NHT resistance has become one of the major obstacles to improving the prognosis of these patients. Therefore, understanding the mechanisms of NHT resistance and being aware of existing and emerging strategies to combat this resistance are fundamental to improving the prognosis of patients with advanced prostate cancer. This review provides a comprehensive overview of the five existing NHT drugs—abiraterone, enzalutamide, apalutamide, darolutamide, and rezvilutamide—by discussing their therapeutic mechanisms, key clinical trials, and treatment side effects. It then summarizes the mechanisms of NHT resistance, which include both AR-dependent and AR-independent pathways. Finally, it reviews both existing and emerging strategies to counteract NHT resistance. Through this systematic summary, we aim to assist clinicians and researchers in better understanding and tackling the therapeutic challenges of advanced prostate cancer.

The mechanisms of resistance to NHT in prostate cancer are complex and multifactorial. Abnormal activation of the AR signaling pathway is the central mechanism of NHT resistance and includes factors such as *AR* amplification, *AR* gene mutations, AR splice variants, increased endogenous androgen synthesis, and post-translational modifications of AR. On the other hand, AR-independent pathways, such as the GR/PR pathway, NEPC, DNA repair defects, autophagy, immune evasion, EMT, PI3K/AKT/mTOR, HGF/MET, and HER2 signaling pathway activation, are also significant contributors to NHT resistance.

Although modest progress has been made in the development of drugs targeting the mechanisms of NHT resistance, no groundbreaking achievements have been realized overall. To address the abnormal activation of the AR signaling pathway, the latest potent AR antagonists—such as apalutamide, darolutamide, and rezvilutamide—show promise in partially overcoming this issue. However, the use of these new drugs needs to be expanded further through large Phase III randomized controlled trials, and their effectiveness as post-NHT strategies after early NHT failure due to cross-resistance also needs further clinical validation. Most other strategies targeting AR pathway resistance mechanisms are still in preclinical models or at the early clinical trial stages and have not yet been widely applied in clinical practice. For AR-independent pathways contributing to NHT resistance, strategies directly targeting resistance mechanisms are mostly still in the preclinical or early clinical trial stages. Overall, current mature clinical strategies to address NHT resistance primarily focus on bypassing rather than directly confronting resistance mechanisms, using additional antitumor mechanisms such as PARPI, chemotherapy, and radionuclide therapy to delay tumor progression. Regarding the future research directions of PARPI, the development of new PARPI will be a key focus to combat PARPI resistance, enhance selectivity, and reduce cytotoxicity, while the exploration of personalized combination therapy strategies involving PARPI will be crucial for enhancing efficacy, and advancements in sequencing technology and the discovery of new biomarkers will aid in identifying more beneficiaries of PARPI treatment. For chemotherapy, resistance remains the most significant challenge, necessitating in-depth research into the molecular mechanisms of chemotherapy resistance, exploration of effective biomarkers, development of combination therapies, optimization of dosage regimens, and provision of supportive care to help reduce the side effects of chemotherapy.

It is noteworthy that immunotherapy and radiotherapy are believed to hold significant potential in advanced prostate cancer, and could represent the types of strategies that may achieve major breakthroughs in the treatment of advanced prostate cancer in the future. Although immunotherapy has achieved significant efficacy in other tumors, the "immune-cold" characteristic of prostate cancer has thus far prevented significant breakthroughs in this area [119]. Future research should focus on identifying prostate cancer patients who could benefit from immunotherapy or on transforming prostate cancer from an 'immune-cold' to a 'hot' tumor [120]. For example, preclinical studies have shown that the PI3K inhibitor BAY1082439 activates the interferon (IFN) α/γ pathway, increases the expression of C-X-C motif chemokine ligand 10 (CXCL10) and C–C motif chemokine ligand 5 (CCL5), inhibits regulatory T cells, and promotes CD8^+^ T cell infiltration, thus alleviating immune suppression in PTEN-deficient prostate cancer [121]. Additionally, studies have shown that combining enzalutamide with the enhancer of zeste homolog 2 (EZH2) inhibitor GSK-126 remodels the tumor immune microenvironment by activating CD8^+^ T cells and IFN-γ production [122].

Previously, radiotherapy was primarily used as palliative treatment to alleviate symptoms in patients with advanced prostate cancer [123]. However, with recent advancements in radiotherapy techniques, its application in advanced prostate cancer has become increasingly widespread. Recent retrospective studies have shown that for patients with oligometastatic mCRPC, radiotherapy targeting oligometastatic sites prolongs the efficacy of NHT, providing a new approach to addressing NHT resistance [124]. According to the phase II clinical trial named EXTEND, which included 87 patients with oligometastatic mCRPC, the results indicated that endocrine therapy combined with radiotherapy prolonged PFS compared to endocrine therapy alone, and the study revealed that only the combination therapy group exhibited T-cell activation, proliferation, and clonal expansion, suggesting that the enhancement of immune modulation by radiotherapy could promote the synergistic effect of endocrine therapy and radiotherapy [125].

Radionuclide therapy, a distinct type of radiotherapy, is making significant strides in the diagnosis and treatment of metastatic prostate cancer. In diagnostics, 68 Ga-labeled PSMA PET imaging exhibits high accuracy, specificity, and sensitivity, which makes it an excellent tool for detecting and evaluating prostate cancer recurrence and metastasis [126]. A study shows that unambiguous radiologic extranodal extension, as determined by MRI, can assist clinical physicians in identifying metastatic prostate cancer patients with an area under the curve value of 0.915 using 68 Ga-PSMA PET/CT. In terms of treatment, radionuclide therapy, using agents such as Ra223 and Lu177, is being utilized in advanced prostate cancer as previously reviewed, with the combination therapy strategies of radionuclides emerging as a key area for future research. Notably, among the existing treatment strategies applied in clinical practice, radionuclide therapy holds the most promise for synergistic effects with immunotherapy. This potential primarily arises from radionuclide therapy's ability to enhance antitumor immune responses by reshaping the TME [127]. As for other combined therapies, combining radionuclide therapy with PARPI or chemotherapy can exert synergistic effects, increasing the cumulative effect of DNA double-strand breaks and interfering with cell division. Additionally, combining Lu177-PSMA with NHT enhances PSMA expression in prostate cancer cells, thereby improving the targeting and efficacy of the radioligand. We eagerly anticipate the results of future clinical trials on these combination strategies involving radionuclide therapy, expected to significantly improve the prognosis of patients with advanced prostate cancer, especially those who have failed NHT.

In addition to the combination strategies involving radionuclide therapy, radiopharmaceutical therapy in prostate cancer presents even more promising avenues for exploration, including new radionuclides, new targets, and new carrier platforms. Regarding new radionuclides, Bi-213, Ac-225, Cu-64, F-18, and Zr-89 exhibit significant potential in the treatment and imaging of prostate cancer. In terms of targets, PSMA remains one of the most promising [128]. Enhancing PSMA-targeted radioligands will improve therapeutic targeting and efficacy [129]. Additionally, exploring other targets like prostate stem cell antigen (PSCA) and integrin αvβ3 contributes to the development of novel radiotherapy strategies [130]. Developing new carrier platforms represents another crucial direction in radiopharmaceutical therapy. The application of nanotechnology enables more precise drug delivery [131], increases the concentration of radiopharmaceuticals at tumor sites [132], and reduces toxic side effects [133]. In summary, radiopharmaceutical therapy holds substantial potential for advancement in the field of advanced prostate cancer and could become an effective post-NHT strategy.

Finally, this discussion addresses why most drugs targeting NHT resistance mechanisms have yet to achieve significant breakthroughs and explores potential directions for future progress. Currently identified NHT resistance mechanisms represent only a subset of the complete spectrum. With advancements in high-resolution sequencing technologies and resistance research models, it is anticipated that additional NHT resistance mechanisms will continue to be uncovered. Cutting-edge technologies such as single-cell sequencing [134,135], spatial transcriptomics [136], proteomics [137], metabolomics [138], and metagenomics are enabling clinicians to utilize clinical samples more effectively to investigate NHT resistance mechanisms in greater depth. These technologies provide unprecedented cellular and molecular insights, revealing tumor cell heterogeneity and dynamic changes within the tumor microenvironment. This knowledge lays the foundation for developing more precise therapeutic targets. For example, single-cell sequencing technology has proven its worth in studies of various tumors. By analyzing the gene expression profiles of individual tumor cells, researchers can identify distinct cell subpopulations and key signaling pathways associated with resistance [139]. In prostate cancer research, single-cell sequencing can reveal intratumoral heterogeneity, aiding in the identification of which cell types develop resistance to NHT and how these cells interact with other components of the tumor microenvironment [140]. Spatial transcriptomics can provide gene expression information from different spatial locations within the tumor tissue, crucial for understanding the behavior of cells in various regions of the tumor microenvironment. In prostate cancer, spatial transcriptomics can help identify differences in resistance mechanisms between the central and peripheral regions of the tumor, thus offering more targeted treatment strategies [141]. Proteomics and metabolomics can provide comprehensive data on protein expression and metabolic states in tumor cells. These data can reveal resistance-related proteins and metabolic pathways, facilitating the development of new biomarkers for early detection of resistance. In prostate cancer, proteomic and metabolomic analyses can identify specific proteins and metabolites linked to NHT resistance, providing a basis for personalized treatment [142]. Metagenomics can comprehensively analyze the microbiome within tumors and their microenvironments. Studies have shown that the microbiome may play a crucial role in tumor resistance [143]. In prostate cancer, metagenomics can help determine whether specific microbial communities are linked to NHT resistance and whether modulating these microbial communities can reverse resistance [144].

Advanced sequencing technologies need ample clinical samples to maximize their effectiveness. Obtaining clinical samples from endocrine therapy-resistant cases, such as CRPC, remains a challenge. The re-biopsy of resistant prostate cancer has yet to be widely adopted in clinical practice and is only featured in a few studies, significantly limiting the application of advanced sequencing technologies in advanced prostate cancer and posing an obstacle to exploring NHT resistance mechanisms [5]. We believe that expanding the avenues for obtaining clinical specimens from advanced prostate cancer, including establishing multi-institutional biobanks, developing non-invasive detection methods, and standardizing prostate re-biopsy procedures, should be a future direction for overcoming NHT resistance.

Resistance research models are also continually advancing through technologies such as 3D bioprinting models and organoid models. By utilizing 3D bioprinting technology, researchers can create detailed tumor tissue models that incorporate diverse cell types and extracellular matrix components [145]. These 3D bioprinting models are particularly useful for studying interactions between cancer cells and their microenvironment, thereby offering a deeper understanding of resistance pathways. Organoid models derived from patient tumors maintain the genetic and phenotypic characteristics of the original tumors, rendering them highly suitable for drug screening and testing personalized treatment plans. In prostate cancer research, organoids can be used to study the efficacy of NHT and identify potential resistance mechanisms [146]. Moreover, combining these advanced models with high-throughput screening techniques will help rapidly test a large number of compounds on models that closely mimic human tumors, thereby accelerating the identification of effective drugs and enhancing the clinical translation potential of preclinical drug research and development [147]. In summary, the application of these advanced models will undoubtedly contribute to the in-depth exploration of NHT resistance mechanisms and the accelerated development of new drugs.

The enhancement of new target identification and drug delivery efficiencies will be two additional breakthroughs in developing drugs targeting NHT resistance mechanisms. Firstly, more advanced sequencing and omics analyses will provide a more comprehensive description of the gene mutations, gene expression changes, and molecular interactions that drive NHT resistance, accelerating the identification of new NHT resistance targets. Additionally, artificial intelligence and machine learning algorithms can analyze large-scale multi-omics data more rapidly and thoroughly, improving the efficiency of resistance target identification [148]. Finally, the use of nanotechnology-based delivery systems can increase the precision and efficiency of drug delivery to resistant prostate cancer cells. Nanoparticles can be designed to transport drugs directly to tumor sites, increasing drug accumulation in resistant prostate cancer cells while minimizing off-target effects [149]. Moreover, nanocarriers can be engineered to release drugs in response to the tumor microenvironment of specific resistant cells, ensuring optimal drug activity at the intended location [150].

## 6 Conclusion

In summary, while NHT have significantly improved the prognosis of patients with advanced prostate cancer, the emergence of resistance continues to pose a substantial challenge. Future basic research should focus on elucidating the molecular mechanisms underlying NHT resistance and identifying novel therapeutic targets. For existing strategies aimed at counteracting NHT resistance, expanding their application, optimizing efficacy, and developing more effective combination approaches should remain key priorities for ongoing research.

## CRediT authorship contribution statement

**Zhipeng Wang:** Writing – review & editing, Writing – original draft, Visualization, Resources, Formal analysis, Data curation, Conceptualization. **Jie Wang:** Writing – review & editing, Writing – original draft, Methodology, Formal analysis, Data curation, Conceptualization. **Dengxiong Li:** Writing – review & editing, Writing – original draft, Visualization, Formal analysis, Data curation, Conceptualization. **Ruicheng Wu:** Writing – review & editing, Writing – original draft, Methodology, Formal analysis, Data curation, Conceptualization. **Jianlin Huang:** Writing – review & editing, Writing – original draft, Data curation, Conceptualization. **Luxia Ye:** Writing – review & editing, Writing – original draft, Methodology. **Zhouting Tuo:** Writing – review & editing, Writing – original draft, Software, Resources. **Qingxin Yu:** Writing – review & editing, Writing – original draft, Resources, Investigation. **Fanglin Shao:** Writing – review & editing, Writing – original draft, Resources. **Dilinaer Wusiman:** Writing – review & editing, Writing – original draft, Visualization, Resources. **William C. Cho:** Writing – review & editing, Validation, Resources, Methodology. **Siang Boon Koh:** Writing – original draft, Supervision, Project administration. **Wei Xiong:** Writing – review & editing, Writing – original draft, Supervision, Project administration. **Dechao Feng:** Writing – review & editing, Writing – original draft, Supervision, Project administration, Investigation.

## Declaration of competing interest

The authors declare that there are no conflicts of interest.

## References

[1] Zi H., Liu M., Luo L. (2024). Global burden of benign prostatic hyperplasia, urinary tract infections, urolithiasis, bladder cancer, kidney cancer, and prostate cancer from 1990 to 2021. Mil. Med. Res..

[2] Zhu Y., Mo M., Wei Y. (2021). Epidemiology and genomics of prostate cancer in Asian men. Nat. Rev. Urol..

[3] Francolini G., Carnevale M.G., Di Cataldo V. (2023). Stereotactic reirradiation with CyberknifeR for locally recurrent prostate cancer, long-term toxicity and clinical outcomes from a monocentric cohort, Radiol. Med.

[4] Li J., Tang T., Zong H. (2024). Intelligent medicine in focus: The 5 stages of evolution in robot-assisted surgery for prostate cancer in the past 20 years and future implications. Mil. Med. Res..

[5] Robinson D., Van Allen E.M., Wu Y. (2015). Integrative clinical genomics of advanced prostate cancer. Cell.

[6] Takeda D.Y., Spisák S., Seo J.H. (2018). A somatically acquired enhancer of the androgen receptor is a noncoding driver in advanced prostate cancer. Cell.

[7] Smith M.R., Hussain M., Saad F. (2022). Darolutamide and survival in metastatic, hormone-sensitive prostate cancer. N. Engl. J. Med..

[8] Moilanen A.M., Riikonen R., Oksala R. (2015). Discovery of ODM-201, a new-generation androgen receptor inhibitor targeting resistance mechanisms to androgen signaling-directed prostate cancer therapies. Sci. Rep..

[9] Mostaghel E.A., Marck B.T., Plymate S.R. (2011). Resistance to CYP17A1 inhibition with abiraterone in castration-resistant prostate cancer: Induction of steroidogenesis and androgen receptor splice variants. Clin. Cancer Res..

[10] Zheng Z., Li J., Liu Y. (2022). The crucial role of AR-V7 in enzalutamide-resistance of castration-resistant prostate cancer. Cancers.

[11] Liu C., Armstrong C., Zhu Y. (2016). Niclosamide enhances abiraterone treatment via inhibition of androgen receptor variants in castration resistant prostate cancer. Oncotarget.

[12] Antonarakis E.S., Lu C., Wang H. (2014). AR-V7 and resistance to enzalutamide and abiraterone in prostate cancer. N. Engl. J. Med..

[13] Mohler J.L., Titus M.A., Bai S. (2011). Activation of the androgen receptor by intratumoral bioconversion of androstanediol to dihydrotestosterone in prostate cancer. Cancer Res..

[14] Chang K.H., Li R., Kuri B. (2013). A gain-of-function mutation in DHT synthesis in castration-resistant prostate cancer. Cell.

[15] Alyamani M., Emamekhoo H., Park S. (2018). HSD3B1(1245A>C) variant regulates dueling abiraterone metabolite effects in prostate cancer. J. Clin. Investig..

[16] Bauman D.R., Steckelbroeck S., Williams M.V. (2006). Identification of the major oxidative 3alpha-hydroxysteroid dehydrogenase in human prostate that converts 5alpha-androstane-3alpha, 17beta-diol to 5alpha-dihydrotestosterone: A potential therapeutic target for androgen-dependent disease. Mol. Endocrinol..

[17] Liu C., Armstrong C.M., Lou W. (2017). Inhibition of AKR1C3 activation overcomes resistance to abiraterone in advanced prostate cancer. Mol. Cancer Ther..

[18] Liu C., Lou W., Zhu Y. (2015). Intracrine androgens and AKR1C3 activation confer resistance to enzalutamide in prostate cancer. Cancer Res..

[19] Liu C., Yang J.C., Armstrong C.M. (2019). AKR1C3 promotes AR-V7 protein stabilization and confers resistance to AR-targeted therapies in advanced prostate cancer. Mol. Cancer Ther..

[20] Zhao J., Zhang M., Liu J. (2019). AKR1C3 expression in primary lesion rebiopsy at the time of metastatic castration-resistant prostate cancer is strongly associated with poor efficacy of abiraterone as a first-line therapy. Prostate.

[21] Venkadakrishnan V.B., Ben-Salem S., Heemers H.V. (2020). AR-dependent phosphorylation and phospho-proteome targets in prostate cancer. Endocr. Relat. Cancer.

[22] Lu D., Song Y., Yu Y. (2021). KAT2A-mediated AR translocation into nucleus promotes abiraterone-resistance in castration-resistant prostate cancer. Cell Death Dis..

[23] Shi L., Yan Y., He Y. (2021). Mutated SPOP E3 ligase promotes 17βHSD4 protein degradation to drive androgenesis and prostate cancer progression. Cancer Res..

[24] Puhr M., Hoefer J., Eigentler A. (2018). The glucocorticoid receptor is a key player for prostate cancer cell survival and a target for improved antiandrogen therapy. Clin. Cancer Res..

[25] Yu Y., Liu L., Xie N. (2013). Expression and function of the progesterone receptor in human prostate stroma provide novel insights to cell proliferation control. J. Clin. Endocrinol. Metab..

[26] Wang Y., Wang Y., Ci X. (2021). Molecular events in neuroendocrine prostate cancer development. Nat. Rev. Urol..

[27] Terry S., Beltran H. (2014). The many faces of neuroendocrine differentiation in prostate cancer progression. Front. Oncol..

[28] Dardenne E., Beltran H., Benelli M. (2016). N-myc induces an EZH2-mediated transcriptional program driving neuroendocrine prostate cancer. Cancer Cell.

[29] Lee J.K., Phillips J.W., Smith B.A. (2016). N-myc drives neuroendocrine prostate cancer initiated from human prostate epithelial cells. Cancer Cell.

[30] De Sarkar N., Dasgupta S., Chatterjee P. (2021). Genomic attributes of homology-directed DNA repair deficiency in metastatic prostate cancer. JCI Insight.

[31] Mateo J., Boysen G., Barbieri C.E. (2017). DNA repair in prostate cancer: Biology and clinical implications. Eur. Urol..

[32] Hientz K., Mohr A., Bhakta-Guha D. (2017). The role of p53 in cancer drug resistance and targeted chemotherapy. Oncotarget.

[33] Mirzayans R., Andrais B., Scott A. (2012). New insights into p53 signaling and cancer cell response to DNA damage: Implications for cancer therapy. J. Biomed. Biotechnol..

[34] Tolkach Y., Kremer A., Lotz G. (2022). Androgen receptor splice variants contribute to the upregulation of DNA repair in prostate cancer. Cancers.

[35] Braglia L., Zavatti M., Vinceti M. (2020). Deregulated PTEN/PI3K/AKT/mTOR signaling in prostate cancer: Still a potential druggable target?. Biochim. Biophys. Acta Mol. Cell Res..

[36] Debnath J., Gammoh N., Ryan K.M. (2023). Autophagy and autophagy-related pathways in cancer. Nat. Rev. Mol. Cell Biol..

[37] Wang Y., Chen J., Wu Z. (2021). Mechanisms of enzalutamide resistance in castration-resistant prostate cancer and therapeutic strategies to overcome it. Br. J. Pharmacol..

[38] Nguyen H.G., Yang J.C., Kung H.J. (2014). Targeting autophagy overcomes Enzalutamide resistance in castration-resistant prostate cancer cells and improves therapeutic response in a xenograft model. Oncogene.

[39] Ziparo E., Petrungaro S., Marini E.S. (2013). Autophagy in prostate cancer and androgen suppression therapy. Int. J. Mol. Sci..

[40] Jeong S.H., Kwak C. (2022). Immunotherapy for prostate cancer: Requirements for a successful regime transfer. Investig. Clin. Urol..

[41] Movassaghi M., Chung R., Anderson C.B. (2021). Overcoming immune resistance in prostate cancer: Challenges and advances. Cancers.

[42] Palicelli A., Croci S., Bisagni A. (2021). What do we have to know about PD-L1 expression in prostate cancer? a systematic literature review. Part 3: PD-L1, intracellular signaling pathways and tumor microenvironment. Int. J. Mol. Sci..

[43] Lamouille S., Xu J., Derynck R. (2014). Molecular mechanisms of epithelial-mesenchymal transition. Nat. Rev. Mol. Cell Biol..

[44] Shiota M., Yokomizo A., Takeuchi A. (2014). Inhibition of protein kinase C/Twist1 signaling augments anticancer effects of androgen deprivation and enzalutamide in prostate cancer. Clin. Cancer Res..

[45] Shiota M., Fujimoto N., Matsumoto T. (2021). Differential impact of TGFB1 variation by metastatic status in androgen-deprivation therapy for prostate cancer. Front. Oncol..

[46] Lin S.R., Yeh H.L., Liu Y.N. (2021). Interplay of epidermal growth factor receptor and signal transducer and activator of transcription 3 in prostate cancer: Beyond androgen receptor transactivation. Cancers.

[47] Peng Y., Wang Y., Zhou C. (2022). PI3K/Akt/mTOR pathway and its role in cancer therapeutics: Are we making headway?. Front. Oncol..

[48] Chen H., Zhou L., Wu X. (2016). The PI3K/AKT pathway in the pathogenesis of prostate cancer. Front. Biosci..

[49] Shorning B.Y., Dass M.S., Smalley M.J. (2020). The PI3K-AKT-mTOR pathway and prostate cancer: At the crossroads of AR, MAPK, and WNT signaling. Int. J. Mol. Sci..

[50] Raghav K.P.S., Moasser M.M. (2023). Molecular pathways and mechanisms of HER2 in cancer therapy. Clin. Cancer Res..

[51] Guerrero J., Alfaro I.E., Gómez F. (2013). Enzalutamide, an androgen receptor signaling inhibitor, induces tumor regression in a mouse model of castration-resistant prostate cancer. Prostate.

[52] Cecchi F., Rabe D.C., Bottaro D.P. (2012). Targeting the HGF/Met signaling pathway in cancer therapy. Expert Opin. Ther. Targets.

[53] Robson S., Pelengaris S., Khan M. (2006). C-Myc and downstream targets in the pathogenesis and treatment of cancer, Recent Pat. Anticancer Drug Discov.

[54] Lin T.P., Li J., Li Q. (2018). R1 regulates prostate tumor growth and progression by transcriptional suppression of the E3 ligase HUWE1 to stabilize c-myc. Mol. Cancer Res..

[55] Solé X., Hernández P., de Heredia M.L. (2008). Genetic and genomic analysis modeling of germline c-MYC overexpression and cancer susceptibility. BMC Genom..

[56] Tripathi A., Supko J.G., Gray K.P. (2020). Dual blockade of c-MET and the androgen receptor in metastatic castration-resistant prostate cancer: A phase I study of concurrent enzalutamide and crizotinib. Clin. Cancer Res..

[57] Bai S., Cao S., Jin L. (2019). A positive role of c-Myc in regulating androgen receptor and its splice variants in prostate cancer. Oncogene.

[58] Guo H., Wu Y., Nouri M. (2021). Androgen receptor and MYC equilibration centralizes on developmental super-enhancer. Nat. Commun..

[59] de Bono J.S., Chowdhury S., Feyerabend S. (2018). Antitumour activity and safety of enzalutamide in patients with metastatic castration-resistant prostate cancer previously treated with abiraterone acetate plus prednisone for ≥24 weeks in Europe. Eur. Urol..

[60] Maughan B.L., Luber B., Nadal R. (2017). Comparing sequencing of abiraterone and enzalutamide in men with metastatic castration-resistant prostate cancer: A retrospective study. Prostate.

[61] Rathkopf D.E., Antonarakis E.S., Shore N.D. (2017). Safety and antitumor activity of apalutamide (ARN-509) in metastatic castration-resistant prostate cancer with and without prior abiraterone acetate and prednisone. Clin. Cancer Res..

[62] Shiota M., Dejima T., Yamamoto Y. (2018). Collateral resistance to taxanes in enzalutamide-resistant prostate cancer through aberrant androgen receptor and its variants. Cancer Sci..

[63] Darshan M.S., Loftus M.S., Thadani-Mulero M. (2011). Taxane-induced blockade to nuclear accumulation of the androgen receptor predicts clinical responses in metastatic prostate cancer. Cancer Res..

[64] Antonarakis E.S., Tagawa S.T., Galletti G. (2017). Randomized, noncomparative, phase II trial of early switch from docetaxel to cabazitaxel or vice versa, with integrated biomarker analysis, in men with chemotherapy-Naïve, metastatic, castration-resistant prostate cancer. J. Clin. Oncol..

[65] Buck S.A.J., Van Hemelryk A., de Ridder C. (2024). Darolutamide added to docetaxel augments antitumor effect in models of prostate cancer through cell cycle arrest at the G1-S transition. Mol. Cancer Ther..

[66] Liu Z., Wei Q., Feng P. (2024). Rezvilutamide (REZ) plus docetaxel (DOC) in patients (pts) with chemo-naïve metastatic castration-resistant prostate cancer (mCRPC) after progression on abiraterone (ABI). J. Clin. Oncol..

[67] Taylor A.K., Kosoff D., Emamekhoo H. (2023). PARP inhibitors in metastatic prostate cancer. Front. Oncol..

[68] Lombard A.P., Liu C., Armstrong C.M. (2019). Overexpressed ABCB1 induces olaparib-taxane cross-resistance in advanced prostate cancer. Transl. Oncol.

[69] Zhu S., Zhao J., Nie L. (2022). Homologous recombination deficiency (HRD) score in aggressive prostatic adenocarcinoma with or without intraductal carcinoma of the prostate (IDC-P). BMC Med..

[70] Li L., Karanika S., Yang G. (2017). Androgen receptor inhibitor-induced “BRCAness” and PARP inhibition are synthetically lethal for castration-resistant prostate cancer. Sci. Signal..

[71] Saad F., Clarke N.W., Oya M. (2023). Olaparib plus abiraterone versus placebo plus abiraterone in metastatic castration-resistant prostate cancer (PROpel): Final prespecified overall survival results of a randomised, double-blind, phase 3 trial. Lancet Oncol..

[72] Chi K.N., Rathkopf D., Smith M.R. (2023). Niraparib and abiraterone acetate for metastatic castration-resistant prostate cancer. J. Clin. Oncol..

[73] Agarwal N., Azad A.A., Carles J. (2023). Talazoparib plus enzalutamide in men with first-line metastatic castration-resistant prostate cancer (TALAPRO-2): A randomised, placebo-controlled, phase 3 trial. Lancet.

[74] Parker C., Nilsson S., Heinrich D. (2013). Alpha emitter Radium-223 and survival in metastatic prostate cancer. N. Engl. J. Med..

[75] Smith M., Parker C., Saad F. (2019). Addition of radium-223 to abiraterone acetate and prednisone or prednisolone in patients with castration-resistant prostate cancer and bone metastases (ERA 223): A randomised, double-blind, placebo-controlled, phase 3 trial. Lancet Oncol..

[76] Maughan B.L., Kessel A., McFarland T.R. (2021). Radium-223 plus enzalutamide versus enzalutamide in metastatic castration-refractory prostate cancer: Final safety and efficacy results. Oncologist.

[77] Connell B., Hwang C., Folefac E. (2024). Fractionated docetaxel (D) and radium 223 (Ra223) in metastatic castration-resistant prostate cancer (CRPC): A modular phase I trial. J. Clin. Oncol..

[78] Pan E., Xie W., Ajmera A. (2023). A phase I study of combination olaparib and Radium-223 in men with metastatic castration-resistant prostate cancer (mCRPC) with bone metastases (COMRADE). Mol. Cancer Ther..

[79] Quinn Z., Leiby B., Sonpavde G. (2023). Phase I study of niraparib in combination with Radium-223 for the treatment of metastatic castrate-resistant prostate cancer. Clin. Cancer Res..

[80] Sartor O., de Bono J., Chi K.N. (2021). Lutetium-177-PSMA-617 for metastatic castration-resistant prostate cancer. N. Engl. J. Med..

[81] Morris M.J., Castellano D., Herrmann K. (2024). 177Lu-PSMA-617 versus a change of androgen receptor pathway inhibitor therapy for taxane-naive patients with progressive metastatic castration-resistant prostate cancer (PSMAfore): A phase 3, randomised, controlled trial. Lancet.

[82] Denmeade S., Antonarakis E.S., Markowski M.C. (2022). Bipolar androgen therapy (BAT): A patient's guide. Prostate.

[83] Denmeade S.R., Wang H., Agarwal N. (2021). Transformer: A randomized phase II study comparing bipolar androgen therapy versus enzalutamide in asymptomatic men with castration-resistant metastatic prostate cancer. J. Clin. Oncol..

[84] Liu C., Armstrong C.M., Lou W. (2017). Niclosamide and bicalutamide combination treatment overcomes enzalutamide- and bicalutamide-resistant prostate cancer. Mol. Cancer Ther..

[85] Myung J.K., Banuelos C.A., Fernandez J.G. (2013). An androgen receptor N-terminal domain antagonist for treating prostate cancer. J. Clin. Investig..

[86] Moon S.J., Jeong B.C., Kim H.J. (2021). Bruceantin targets HSP90 to overcome resistance to hormone therapy in castration-resistant prostate cancer. Theranostics.

[87] Liu Z., Hu M., Yang Y. (2022). An overview of PROTACs: A promising drug discovery paradigm. Mol. Biomed..

[88] Chen Q., Munoz E., Ashong D. (2024). Insight into recent advances in degrading androgen receptor for castration-resistant prostate cancer. Cancers.

[89] Petrylak D.P., Gao X., Vogelzang N.J. (2020). First-in-human phase I study of ARV-110, an androgen receptor (AR) PROTAC degrader in patients (pts) with metastatic castrate-resistant prostate cancer (mCRPC) following enzalutamide (ENZ) and/or abiraterone (ABI). J. Clin. Oncol..

[90] Petrylak D.P., Stewart T.F., Gao X. (2023). A phase 2 expansion study of ARV-766, a PROTAC androgen receptor (AR) degrader, in metastatic castration-resistant prostate cancer (mCRPC). J. Clin. Oncol..

[91] Petrylak D.P., Garmezy B., Shen J. (2023). 1803P Phase I/II study of bavdegalutamide, a PROTAC androgen receptor (AR) degrader in metastatic castration-resistant prostate cancer (mCRPC): Radiographic progression-free survival (rPFS) in patients (pts) with AR ligand-binding domain (LBD) mutations. Ann. Oncol..

[92] Rathkopf D.E., Patel M.R., Choudhury A.D. (2025). Safety and clinical activity of BMS-986365 (CC-94676), a dual androgen receptor ligand-directed degrader and antagonist, in heavily pretreated patients with metastatic castration-resistant prostate cancer. Ann. Oncol..

[93] Isikbay M., Otto K., Kregel S. (2014). Glucocorticoid receptor activity contributes to resistance to androgen-targeted therapy in prostate cancer, Horm. Cancer.

[94] Serritella A.V., Shevrin D., Heath E.I. (2022). Phase I/II trial of enzalutamide and mifepristone, a glucocorticoid receptor antagonist, for metastatic castration-resistant prostate cancer. Clin. Cancer Res..

[95] Meresse P., Dechaux E., Monneret C. (2004). Etoposide: Discovery and medicinal chemistry. Curr. Med. Chem..

[96] de Kouchkovsky I., Chan E., Schloss C. (2024). Diagnosis and management of neuroendocrine prostate cancer. Prostate.

[97] Rocha C.R.R., Silva M.M., Quinet A. (2018). DNA repair pathways and cisplatin resistance: An intimate relationship. Clinics.

[98] Das C.M., Zage P.E., Taylor P. (2010). Chromatin remodelling at the topoisomerase II-beta promoter is associated with enhanced sensitivity to etoposide in human neuroblastoma cell lines. Eur. J. Cancer.

[99] Mosca L., Ilari A., Fazi F. (2021). Taxanes in cancer treatment: Activity, chemoresistance and its overcoming. Drug Resist. Updat..

[100] Alabi B.R., Liu S., Stoyanova T. (2022). Current and emerging therapies for neuroendocrine prostate cancer. Pharmacol. Ther..

[101] Hansen A., Massard C., Ott P.A. (2016). Pembrolizumab for patients with advanced prostate adenocarcinoma: Preliminary results from the KEYNOTE-028 study. Ann. Oncol..

[102] Antonarakis E.S., Piulats J.M., Gross-Goupil M. (2021). 611P Pembrolizumab (pembro) monotherapy for docetaxel-pretreated metastatic castration-resistant prostate cancer (mCRPC): Updated analyses with 4 years of follow-up from cohorts 1-3 of the KEYNOTE-199 study. Ann. Oncol..

[103] Graff J.N., Burotto M., Fong P.C. (2023). 1771MO Pembrolizumab (pembro) plus enzalutamide (enza) for patients (pts) with metastatic castration-resistant prostate cancer (mCRPC): Randomized double-blind phase III KEYNOTE-641 study. Ann. Oncol..

[104] Petrylak D., Ratta R., Matsubara N. (2023). Pembrolizumab plus docetaxel for patients with metastatic castration-resistant prostate cancer (mCRPC): Randomized, double-blind, phase 3 KEYNOTE-921 study. J. Clin. Oncol..

[105] Antonarakis E.S., Park S.H., Goh J.C. (2023). Pembrolizumab plus olaparib for patients with previously treated and biomarker-unselected metastatic castration-resistant prostate cancer: The randomized, open-label, phase III KEYLYNK-010 trial. J. Clin. Oncol..

[106] Fizazi K., Retz M., Petrylak D.P. (2022). Nivolumab plus rucaparib for metastatic castration-resistant prostate cancer: Results from the phase 2 CheckMate 9KD trial. J. Immunother. Cancer.

[107] Fizazi K., González Mella P., Castellano D. (2022). Nivolumab plus docetaxel in patients with chemotherapy-naïve metastatic castration-resistant prostate cancer: Results from the phase II CheckMate 9KD trial. Eur. J. Cancer.

[108] Sweeney C.J., Gillessen S., Rathkopf D. (2020). Abstract CT014: IMbassador250: A phase III trial comparing atezolizumab with enzalutamide vs enzalutamide alone in patients with metastatic castration-resistant prostate cancer (mCRPC). Cancer Res..

[109] Sharma P., Pachynski R.K., Narayan V. (2020). Nivolumab plus ipilimumab for metastatic castration-resistant prostate cancer: Preliminary analysis of patients in the CheckMate 650 trial. Cancer Cell.

[110] Liu Q., Tong D., Liu G. (2017). Metformin reverses prostate cancer resistance to enzalutamide by targeting TGF-β1/STAT3 axis-regulated EMT. Cell Death Dis..

[111] Qi W., Morales C., Cooke L.S. (2015). Reciprocal feedback inhibition of the androgen receptor and PI3K as a novel therapy for castrate-sensitive and-resistant prostate cancer. Oncotarget.

[112] Sugawara T., Nevedomskaya E., Heller S. (2024). Dual targeting of the androgen receptor and PI3K/AKT/mTOR pathways in prostate cancer models improves antitumor efficacy and promotes cell apoptosis. Mol. Oncol..

[113] Sweeney C., Bracarda S., Sternberg C.N. (2021). Ipatasertib plus abiraterone and prednisolone in metastatic castration-resistant prostate cancer (IPATential150): A multicentre, randomised, double-blind, phase 3 trial. Lancet.

[114] Zhao J.L., Antonarakis E.S., Cheng H.H. (2024). Phase 1b study of enzalutamide plus CC-115, a dual mTORC1/2 and DNA-PK inhibitor, in men with metastatic castration-resistant prostate cancer (mCRPC). Br. J. Cancer.

[115] Sweeney C.J., Percent I.J., Babu S. (2022). Phase ib/II study of enzalutamide with samotolisib (LY3023414) or placebo in patients with metastatic castration-resistant prostate cancer. Clin. Cancer Res..

[116] Asangani I.A., Wilder-Romans K., Dommeti V.L. (2016). BET bromodomain inhibitors enhance efficacy and disrupt resistance to AR antagonists in the treatment of prostate cancer. Mol. Cancer Res..

[117] Shiota M., Bishop J.L., Takeuchi A. (2015). Inhibition of the HER2-YB1-AR axis with Lapatinib synergistically enhances Enzalutamide anti-tumor efficacy in castration resistant prostate cancer. Oncotarget.

[118] Verras M., Lee J., Xue H. (2007). The androgen receptor negatively regulates the expression of c-Met: Implications for a novel mechanism of prostate cancer progression. Cancer Res..

[119] Stultz J., Fong L. (2021). How to turn up the heat on the cold immune microenvironment of metastatic prostate cancer. Prostate Cancer Prostatic Dis..

[120] Runcie K.D., Dallos M.C. (2021). Prostate cancer immunotherapy-finally in from the cold?. Curr. Oncol. Rep..

[121] Qi Z., Xu Z., Zhang L. (2022). Overcoming resistance to immune checkpoint therapy in PTEN-null prostate cancer by intermittent anti-PI3Kα/β/δ treatment. Nat. Commun..

[122] Fischetti I., Botti L., Sulsenti R. (2024). Combined therapy targeting AR and EZH2 curbs castration-resistant prostate cancer enhancing anti-tumor T-cell response. Epigenomics.

[123] Boyer M.J., Salama J.K., Robert Lee W. (2014). Palliative radiotherapy for prostate cancer. Oncology.

[124] Valeriani M., Detti B., Fodor A. (2022). Radiotherapy at oligoprogression for metastatic castration-resistant prostate cancer patients: A multi-institutional analysis. Radiol. Med..

[125] Tang C., Sherry A.D., Haymaker C. (2023). Addition of metastasis-directed therapy to intermittent hormone therapy for oligometastatic prostate cancer: The EXTEND phase 2 randomized clinical trial. JAMA Oncol..

[126] Zhao G., Ji B. (2022). Head-to-head comparison of 68Ga-PSMA-11 PET/CT and 99mTc-MDP bone scintigraphy for the detection of bone metastases in patients with prostate cancer: A meta-analysis. AJR Am. J. Roentgenol..

[127] Sun Q., Li J., Ding Z. (2023). Radiopharmaceuticals heat anti-tumor immunity. Theranostics.

[128] Jang A., Kendi A.T., Sartor O. (2023). Status of PSMA-targeted radioligand therapy in prostate cancer: Current data and future trials. Ther. Adv. Med. Oncol..

[129] Parghane R.V., Basu S. (2023). PSMA-targeted radioligand therapy in prostate cancer: Current status and future prospects. Expert Rev. Anticancer Ther..

[130] Li D., Xu W., Chang Y. (2023). Advances in landscape and related therapeutic targets of the prostate tumor microenvironment. Acta Biochim. Biophys. Sin..

[131] Ma J., Li N., Wang J. (2023). In vivo synergistic tumor therapies based on copper sulfide photothermal therapeutic nanoplatforms. Exploration.

[132] Tian M., Xin X., Wu R. (2022). Advances in intelligent-responsive nanocarriers for cancer therapy. Pharmacol. Res..

[133] Wang A.Z., Tepper J.E. (2014). Nanotechnology in radiation oncology. J. Clin. Oncol..

[134] Ya X., Li H., Ge P. (2024). Single-cell atlas of atherosclerosis patients by cytof: Circulatory and local immune disorders. Aging Dis.

[135] Hou Y., Yao H., Lin J. (2023). Recent advancements in single-cell metabolic analysis for pharmacological research. J. Pharm. Anal..

[136] Li D., Fang Z., Shi Q. (2023). Single-cell RNA-sequencing and subcellular spatial transcriptomics facilitate the translation of liver microphysiological systems for regulatory application. J. Pharm. Anal..

[137] Yi G., Luo H., Zheng Y. (2025). Exosomal proteomics: Unveiling novel insights into lung cancer. Aging Dis..

[138] Sun Q., Xu Q., Wang M. (2023). OpenNAU: An open-source platform for normalizing, analyzing, and visualizing cancer untargeted metabolomics data. Chin. J. Cancer Res..

[139] Iida K., Okada M. (2024). Identifying key regulatory genes in drug resistance acquisition: Modeling pseudotime trajectories of breast cancer single-cell transcriptome. Cancers.

[140] Song H., Weinstein H.N.W., Allegakoen P. (2022). Single-cell analysis of human primary prostate cancer reveals the heterogeneity of tumor-associated epithelial cell states. Nat. Commun..

[141] Zhang A., Miao K., Sun H. (2022). Tumor heterogeneity reshapes the tumor microenvironment to influence drug resistance. Int. J. Biol. Sci..

[142] Goo Y.A., Goodlett D.R. (2010). Advances in proteomic prostate cancer biomarker discovery. J. Proteomics.

[143] Zhou Y., Han W., Feng Y. (2024). Microbial metabolites affect tumor progression, immunity and therapy prediction by reshaping the tumor microenvironment (Review). Int. J. Oncol..

[144] Lebron A.I.C., Balbuena-Almodóvar P.A., Mummert L. (2024). Abstract 3572: Role of the gut microbiome in androgen production and prostate cancer treatment resistance. Cancer Res..

[145] Shukla P., Yeleswarapu S., Heinrich M.A. (2022). Mimicking tumor microenvironment by 3D bioprinting: 3D cancer modeling. Biofabrication.

[146] Waseem M., Wang B.D. (2024). Organoids: An emerging precision medicine model for prostate cancer research. Int. J. Mol. Sci..

[147] Giri A.K., Ianevski A. (2022). High-throughput screening for drug discovery targeting the cancer cell-microenvironment interactions in hematological cancers. Expert Opin. Drug Discov..

[148] Wei L., Niraula D., Gates E.D.H. (2023). Artificial intelligence (AI) and machine learning (ML) in precision oncology: A review on enhancing discoverability through multiomics integration. Br. J. Radiol..

[149] Garbayo E., Pascual-Gil S., Rodríguez-Nogales C. (2020). Nanomedicine and drug delivery systems in cancer and regenerative medicine. Wires Nanomed. Nanobiotechnol..

[150] Sun T., Zhang Y.S., Pang B. (2014). Engineered nanoparticles for drug delivery in cancer therapy. Angew. Chem. Int. Ed..

