# Supplementary material for: Novel hormone therapies for advanced prostate cancer: Understanding and countering drug resistance
Source: J Pharm Anal. 2025 Feb 22;15(9):101232. doi: 10.1016/j.jpha.2025.101232 (PMC12512997; doi:10.1016/j.jpha.2025.101232)
Supplement: Multimedia component 1 [file mmc1.docx]

- 1. **Abiraterone acetate**

Abiraterone acetate functions by inhibiting the enzyme cytochrome P450 17A1 (CYP17A1)[1]. Located in the endoplasmic reticulum, CYP17A1 catalyzes the conversion of pregnenolone to dehydroepiandrosterone (DHEA), a critical step in androgen synthesis[2]. As a CYP17A1 inhibitor, abiraterone significantly reduces androgen synthesis. The Food and Drug Administration (FDA) approved abiraterone acetate in 2011, based primarily on the positive outcomes of two pivotal Phase III RCTs, namely COU-AA-301 and COU-AA-302. The COU-AA-301 trial targeted mCRPC patients post-first-line chemotherapy failure, enrolling 1195 participants. It demonstrated significant extensions in prostate-specific antigen progression-free survival (PSA-PFS), radiographic progression-free survival (rPFS), and overall survival (OS) for the abiraterone group compared to the placebo group (median PSA-PFS: 8.5 months vs. 6.6 months, P<0.001; median rPFS: 5.6 months vs. 3.6 months, P<0.001; median OS: 15.8 months vs. 11.2 months, P<0.001)[3]. Similarly, the COU-AA-302 trial, focusing on mCRPC patients without prior chemotherapy, involved 1088 participants and reported significant prolongations of rPFS and OS in the abiraterone treatment group (median rPFS: 16.5 months vs. 8.2 months, P<0.001; median OS: 34.7 months vs. 30.3 months, P=0.003)[4, 5].

The success of abiraterone in treating mCRPC prompted further investigations into its efficacy for mHSPC. The LATITUDE study targeted high-risk mHSPC patients—defined as at least two of the three factors: Gleason score ≥8, more than three bone metastases, and visceral metastasis—and involved 1199 participants. Results demonstrated significant extensions in rPFS and OS for the abiraterone treatment group compared to controls (median rPFS: 33.0 months vs. 14.8 months, P<0.001; median OS: 53.3 months vs. 36.5 months, P<0.001)[6, 7]. Another study, STAMPEDE, which enrolled 1917 patients with locally advanced prostate cancer or mHSPC, found that abiraterone significantly improved the 3-year OS rate (83% vs. 76%, P<0.001) and the 3-year disease PFS rate (75% vs. 45%, P<0.001)[8].

Potential side effects of abiraterone treatment include fluid retention, hypokalemia, and hypertension. These effects primarily result from its simultaneous inhibition of androgen synthesis and its impact on glucocorticoid synthesis, which enhances feedback in the hypothalamic-pituitary-adrenal axis. This feedback stimulates the secretion of adrenal cortex hormones, which in turn leads to excessive mineralocorticoid secretion, triggering related side effects[9]. Consequently, corticosteroids such as prednisone are frequently co-administered with abiraterone in clinical practice to manage these side effects and enhance the safety and tolerance of the treatment.

- 1. **Enzalutamide**

Enzalutamide, a novel non-steroidal AR antagonist, exhibits a binding affinity for the AR that is approximately five to eight times greater than that of first-generation antiandrogens such as bicalutamide. Enzalutamide exerts its antitumor effects by selectively antagonizing the AR, inhibiting the nuclear translocation of the AR, blocking the recruitment of AR co-activators, and preventing the AR from binding with DNA[10]. Enzalutamide has received FDA approval for use in various stages of prostate cancer, including nmCRPC, mHSPC, and mCRPC.

The clinical application of enzalutamide in the mCRPC stage primarily relies on the outcomes of two Phase III RCTs, AFFIRM and PREVAIL. The AFFIRM trial, involving 1199 patients who had failed first-line chemotherapy for mCRPC, indicated significant improvements in PSA-PFS, rPFS, and OS for the enzalutamide group compared to the placebo group (median PSA-PFS: 8.3 months vs. 3.0 months, P<0.001; median rPFS: 8.3 months vs. 2.9 months, P<0.001; median OS: 18.4 months vs. 13.6 months, P<0.001)[11]. Similarly, the PREVAIL trial enrolled 1,717 mCRPC patients without prior chemotherapy, demonstrating significant extensions in rPFS and OS for the enzalutamide group compared to placebo (median rPFS: not reached vs. 3.9 months, P<0.001; median OS: 32.4 months vs. 30.2 months, P<0.001)[12].

Following the successful outcomes of the ARCHES and ENZAMET trials, indications for enzalutamide were expanded to include mHSPC patients. In the ARCHES trial, which included 1150 mHSPC patients, the enzalutamide treatment group exhibited significantly longer rPFS and OS (median rPFS: 49.8 vs. 38.9 months, P<0.001; median OS: not reach (NR) vs. NR, P<0.001)[13, 14]. The ENZAMET trial revealed enzalutamide's superior efficacy over first-generation antiandrogens, reducing the risk of death by 30% and increasing the five-year survival rate from 57% to 67%, significantly prolong the PSA-PFS, clinical PFS, and OS (median PSA-PFS: 68.0 vs. 22.0 months, P<0.001; median clinical PFS: 81.0 months vs. 25.0 months, P<0.001; median OS: NR vs. NR, P<0.001)[15, 16].

The PROSPER trial laid the groundwork for the use of enzalutamide in the nmCRPC stage[17] . Involving 1401 nmCRPC patients, the study demonstrated that enzalutamide significantly extended metastasis-free survival (MFS), PSA-PFS, and OS (median MFS: 36.6 months vs. 14.7 months, P<0.001; median PSA-PFS: 37.2 months vs. 3.9 months, P<0.001; median OS: 67.0 months vs. 56.3 months, P=0.001) compared to placebo[18].

The primary side effects of enzalutamide, including fatigue, hot flashes, and musculoskeletal pain, are generally associated with AR inhibition and decreased androgen levels[19]. Most patients manage these side effects effectively with appropriate symptomatic treatment. However, it is important to note that a small percentage of patients may experience seizures after receiving enzalutamide, likely because the drug crosses the blood-brain barrier and impacts the central nervous system[20].

- 1. **Apalutamide**

Similar to enzalutamide, apalutamide is a novel AR antagonist. Apalutamide operates by selectively antagonizing the AR, blocking its nuclear translocation, inhibiting the recruitment of AR co-factors, and preventing AR-DNA binding, which contributes to its substantial anti-tumor activity[21]. Its binding affinity for the AR is seven to ten times greater than that of first-generation anti-androgen drugs such as bicalutamide. Apalutamide effectively inhibits AR activity even in cases of AR overexpression or the presence of the *AR* F877L mutation, demonstrating its potential to treat prostate cancers characterized by aberrant AR activation or mutations [21, 22]. Apalutamide is FDA-approved for the treatment of mHSPC and nmCRPC patients.

The approval of apalutamide for mHSPC was primarily based on the findings from the TITAN Phase III RCT [23]. In the TITAN study, involving 1052 mHSPC patients, apalutamide significantly reduced the risk of death by 35% (median time: not reached vs. 52.2 months, P<0.001) and delayed the progression of mHSPC to mCRPC (median time: NR vs. 11.4 months, P<0.001) compared to placebo [24]. Another Phase III clinical trial, the SPARTAN trial, confirmed the efficacy of apalutamide in nmCRPC patients. The SPARTAN trial, involving 1207 participants, demonstrated that apalutamide significantly extended MFS and PSA-PFS (median MFS: 40.5 months vs. 16.2 months, P<0.001; median PSA-PFS: not reached vs. 3.7 months, P<0.001) compared to placebo[25].

Although no large-scale Phase III clinical trials have validated apalutamide's use in mCRPC patients, several studies highlight its potential in this context. In a Phase II clinical trial, mCRPC patients not previously treated with abiraterone achieved a median PSA-PFS of 18.2 months with apalutamide treatment, while those previously treated with abiraterone attained a median PSA-PFS of 3.7 months[26]. The ACIS Phase III clinical trial, which included 982 mCRPC patients, showed that combining abiraterone with apalutamide significantly extended rPFS compared to abiraterone alone (median time: 24.0 months vs. 16.6 months, P<0.001)[27].

Studies suggest that apalutamide achieves maximum tumor treatment efficacy at lower doses and maintains lower concentrations in the central nervous system, potentially reducing neurological side effects[22]. Common adverse effects associated with AR antagonists, such as fatigue, hot flashes, and musculoskeletal pain, are prevalent with apalutamide, which additionally poses risks for rash, pruritus, and hypothyroidism, all necessitating vigilant monitoring. Most patients can effectively manage these side effects with appropriate clinical management.

- 1. **Darolutamide**

Darolutamide, along with enzalutamide and apalutamide, is classified as a novel AR antagonist. Although darolutamide shares a similar therapeutic mechanism with enzalutamide and apalutamide, it has a unique molecular structure and distinct pharmacological properties[28]. A notable characteristic of darolutamide is its ability to inhibit *AR* mutants, including *AR* T877A, *AR* W742L, and *AR* F876L, which is crucial for overcoming resistance to drugs such as bicalutamide, flutamide, enzalutamide, and apalutamide[29]. Therefore, darolutamide shows significant potential in treating AR-targeted drug resistance.

Darolutamide was approved for treating nmCRPC patients, based primarily on the positive results of the Phase III RCT, ARAMIS. In the ARAMIS trial, which enrolled 1509 nmCRPC patients, darolutamide significantly improved MFS (median MFS: 40.4 months vs. 18.4 months, P<0.001) and 3-yr OS rate (83% vs. 77%; hazard ratio (HR): 0.69; 95% confidence interval (CI): 0.53-0.88; P=0.003) compared to placebo, and showed significant benefits across other secondary endpoints, including pain progression time, time to chemotherapy, and time to symptomatic skeletal events[30].

The ARASENS trial explored the effectiveness of combination of darolutamide and docetaxel in mHSPC. In this Phase III clinical trial involving 1306 mHSPC patients, compared to docetaxel, the combination of darolutamide and docetaxel significantly improved patients’ OS and castration resistant-free survival (CFS) (median OS: not reached vs. 48.9 months, P<0.001; median CFS: not reached vs. 19.1 months, P<0.001)[31]. The FDA has officially approved the combination of darolutamide and docetaxel for the treatment of mHSPC. It is noteworthy that the latest Phase III RCT—the ARANOTE trial—has reported the efficacy of darolutamide without concurrent docetaxel in mHSPC[32]. This study included 669 patients with mHSPC, demonstrating that darolutamide significantly improved rPFS by 46% compared to placebo (HR: 0.54; 95% CI, 0.41-0.71; P<0.001). Furthermore, darolutamide showed benefits in other secondary endpoints such as CFS and PSA-PFS. With the ongoing disclosure of further results from this study, the future application of darolutamide in the mHSPC stage is anticipated to expand further.

In addition to typical adverse reactions to AR antagonists such as fatigue, hypertension, and nausea, darolutamide is associated with graver concerns, including cardiac arrhythmia, heart failure, and coronary artery disorders. Consequently, for patients presenting with clinically significant cardiovascular conditions—specifically stroke, myocardial infarction, angina, and heart failure—vigorous management of these primary cardiovascular diseases is imperative when prescribing darolutamide. Additionally, due to darolutamide’s limited penetration into the central nervous system, darolutamide is less likely to cause central nervous system-related side effects compared to enzalutamide[30].

- 1. **Rezvilutamide**

Rezvilutamide, previously known as SHR3680, is a novel AR antagonist with a therapeutic mechanism similar to that of enzalutamide. Developed and clinically tested primarily in China, rezvilutamide has received approval from the National Medical Products Administration of China for treating mHSPC.

Initially designed for mCRPC patients, rezvilutamide was evaluated in Phase I/II clinical trials involving 197 participants. After 12 weeks of treatment, rezvilutamide yielded a PSA response in 68% of patients, stabilized bone disease in 88%, and elicited responses in soft tissue lesions in 34%, with only 11.7% experiencing Grade ≥3 treatment-related adverse events (TRAE)[33]​​. These results confirmed rezvilutamide's efficacy and safety in mCRPC patients, leading to the initiation of the Phase III CHART clinical trial. In the CHART trial, involving 654 patients with high-volume mHSPC, rezvilutamide significantly reduced progression risk by 56% and death risk by 42%, markedly extending rPFS and OS than bicalutamide (median rPFS: NR vs. 25.1 months, P<0.001; median OS: NR vs. NR, P<0.001)[34].

Beyond the typical adverse reactions associated with AR antagonists, hepatotoxicity emerges as a particularly alarming concern during treatment with rezvilutamide. It is imperative to exercise caution when administering rezvilutamide to patients with moderate to severe hepatic impairment, necessitating regular monitoring of liver function to mitigate potential risks. Notably, the low blood-brain barrier permeability of rezvilutamide means it has a significantly lower penetration rate than enzalutamide, significantly reducing the risk of inducing seizures[33].

**Reference：**

[1] C. Thoma, Pharmacology: How abiraterone works, Nat. Rev. Urol. 12 (2015), 363.

[2] D. Porubek, CYP17A1: A biochemistry, chemistry, and clinical review, Curr. Top. Med. Chem. 13 (2013) 1364–1384.

[3] K. Fizazi, H.I. Scher, A. Molina, et al., Abiraterone acetate for treatment of metastatic castration-resistant prostate cancer: Final overall survival analysis of the COU-AA-301 randomised, double-blind, placebo-controlled phase 3 study, Lancet Oncol. 13 (2012) 983–992.

[4] C.J. Ryan, M.R. Smith, K. Fizazi, et al., Abiraterone acetate plus prednisone versus placebo plus prednisone in chemotherapy-naive men with metastatic castration-resistant prostate cancer (COU-AA-302): Final overall survival analysis of a randomised, double-blind, placebo-controlled phase 3 study, Lancet Oncol. 16 (2015) 152–160.

[5] D.E. Rathkopf, M.R. Smith, J.S. de Bono, et al., Updated interim efficacy analysis and long-term safety of abiraterone acetate in metastatic castration-resistant prostate cancer patients without prior chemotherapy (COU-AA-302), Eur. Urol. 66 (2014) 815–825.

[6] K. Fizazi, N. Tran, L. Fein, et al., Abiraterone plus prednisone in metastatic, castration-sensitive prostate cancer, N. Engl. J. Med. 377 (2017) 352–360.

[7] K. Fizazi, N. Tran, L. Fein, et al., Abiraterone acetate plus prednisone in patients with newly diagnosed high-risk metastatic castration-sensitive prostate cancer (LATITUDE): Final overall survival analysis of a randomised, double-blind, phase 3 trial, Lancet Oncol. 20 (2019) 686–700.

[8] N.D. James, J.S. de Bono, M.R. Spears, et al., Abiraterone for prostate cancer not previously treated with hormone therapy, N. Engl. J. Med. 377 (2017) 338–351.

[9] G. Sonpavde, G. Attard, J. Bellmunt, et al., The role of abiraterone acetate in the management of prostate cancer: A critical analysis of the literature, Eur. Urol. 60 (2011) 270–278.

[10] C. Tran, S. Ouk, N.J. Clegg, et al., Development of a second-generation antiandrogen for treatment of advanced prostate cancer, Science 324 (2009) 787–790.

[11] H.I. Scher, K. Fizazi, F. Saad, et al., Increased survival with enzalutamide in prostate cancer after chemotherapy, N. Engl. J. Med. 367 (2012) 1187–1197.

[12] T.M. Beer, A.J. Armstrong, D.E. Rathkopf, et al., Enzalutamide in metastatic prostate cancer before chemotherapy, N. Engl. J. Med. 371 (2014) 424–433.

[13] A.J. Armstrong, R.Z. Szmulewitz, D.P. Petrylak, et al., ARCHES: A randomized, phase III study of androgen deprivation therapy with enzalutamide or placebo in men with metastatic hormone-sensitive prostate cancer, J. Clin. Oncol. 37 (2019) 2974–2986.

[14] A.J. Armstrong, A.A. Azad, T. Iguchi, et al., Improved survival with enzalutamide in patients with metastatic hormone-sensitive prostate cancer, J. Clin. Oncol. 40 (2022) 1616–1622.

[15] I.D. Davis, A.J. Martin, M.R. Stockler, et al., Enzalutamide with standard first-line therapy in metastatic prostate cancer, N. Engl. J. Med. 381 (2019) 121–131.

[16] C.J. Sweeney, A.J. Martin, M.R. Stockler, et al., Testosterone suppression plus enzalutamide versus testosterone suppression plus standard antiandrogen therapy for metastatic hormone-sensitive prostate cancer (ENZAMET): An international, open-label, randomised, phase 3 trial, Lancet Oncol. 24 (2023) 323–334.

[17] M. Hussain, K. Fizazi, F. Saad, et al., Enzalutamide in men with nonmetastatic, castration-resistant prostate cancer, N. Engl. J. Med. 378 (2018) 2465–2474.

[18] C.N. Sternberg, K. Fizazi, F. Saad, et al., Enzalutamide and survival in nonmetastatic, castration-resistant prostate cancer, N. Engl. J. Med. 382 (2020) 2197–2206.

[19] J.N. Graff, M.J. Gordon, T.M. Beer, Safety and effectiveness of enzalutamide in men with metastatic, castration-resistant prostate cancer, Expert Opin. Pharmacother. 16 (2015) 749–754.

[20] D. Pilon, A.S. Behl, L.A. Ellis, et al., Assessment of real-world central nervous system events in patients with advanced prostate cancer using abiraterone acetate, bicalutamide, enzalutamide, or chemotherapy, Am. Health Drug Benefits 10 (2017) 143–153.

[21] J.T. Chong, W.K. Oh, B.C. Liaw, Profile of apalutamide in the treatment of metastatic castration-resistant prostate cancer: Evidence to date, Onco. Targets Ther. 11 (2018) 2141–2147.

[22] N.J. Clegg, J. Wongvipat, J.D. Joseph, et al., ARN-509: A novel antiandrogen for prostate cancer treatment, Cancer Res. 72 (2012) 1494–1503.

[23] K.N. Chi, N. Agarwal, A. Bjartell, et al., Apalutamide for metastatic, castration-sensitive prostate cancer, N. Engl. J. Med. 381 (2019) 13–24.

[24] K.N. Chi, S. Chowdhury, A. Bjartell, et al., Apalutamide in patients with metastatic castration-sensitive prostate cancer: Final survival analysis of the randomized, double-blind, phase III TITAN study, J. Clin. Oncol. 39 (2021) 2294–2303.

[25] M.R. Smith, F. Saad, S. Chowdhury, et al., Apalutamide treatment and metastasis-free survival in prostate cancer, N. Engl. J. Med. 378 (2018) 1408–1418.

[26] D.E. Rathkopf, E.S. Antonarakis, N.D. Shore, et al., Safety and antitumor activity of apalutamide (ARN-509) in metastatic castration-resistant prostate cancer with and without prior abiraterone acetate and prednisone, Clin. Cancer Res. 23 (2017) 3544–3551.

[27] F. Saad, E. Efstathiou, G. Attard, et al., Apalutamide plus abiraterone acetate and prednisone versus placebo plus abiraterone and prednisone in metastatic, castration-resistant prostate cancer (ACIS): A randomised, placebo-controlled, double-blind, multinational, phase 3 study, Lancet Oncol. 22 (2021) 1541–1559.

[28] N.D. Shore, Darolutamide (ODM-201) for the treatment of prostate cancer, Expert Opin. Pharmacother. 18 (2017) 945–952.

[29] A.M. Moilanen, R. Riikonen, R. Oksala, et al., Discovery of ODM-201, a new-generation androgen receptor inhibitor targeting resistance mechanisms to androgen signaling-directed prostate cancer therapies, Sci. Rep. 5 (2015), 12007.

[30] K. Fizazi, N. Shore, T.L. Tammela, et al., Darolutamide in nonmetastatic, castration-resistant prostate cancer, N. Engl. J. Med. 380 (2019) 1235–1246.

[31] M.R. Smith, M. Hussain, F. Saad, et al., Darolutamide and survival in metastatic, hormone-sensitive prostate cancer, N. Engl. J. Med. 386 (2022) 1132–1142.

[32] F. Saad, E. Vjaters, N. Shore, et al., Darolutamide in combination with androgen-deprivation therapy in patients with metastatic hormone-sensitive prostate cancer from the phase III ARANOTE trial, J. Clin. Oncol. 42 (2024) 4271–4281.

[33] X. Qin, D. Ji, W. Gu, et al., Activity and safety of SHR3680, a novel antiandrogen, in patients with metastatic castration-resistant prostate cancer: A phase I/II trial, BMC Med. 20 (2022), 84.

[34] W. Gu, W. Han, H. Luo, et al., Rezvilutamide versus bicalutamide in combination with androgen-deprivation therapy in patients with high-volume, metastatic, hormone-sensitive prostate cancer (CHART): A randomised, open-label, phase 3 trial, Lancet Oncol. 23 (2022) 1249–1260.
