# Supplementary material for: Novel hormone therapies for advanced prostate cancer: Understanding and countering drug resistance
Source: J Pharm Anal. 2025 Feb 22;15(9):101232. doi: 10.1016/j.jpha.2025.101232 (PMC12512997; doi:10.1016/j.jpha.2025.101232)
Supplement: Multimedia component 2 [file mmc2.docx]

| Supplementary table 1. Distinct properties of novel hormone drugs. | | | | |
| --- | --- | --- | --- | --- |
| Drugs | **Pharmacological action** | **Appropriate disease stages and Phase III RCT evidence** | **Specific AEs** | **Other characteristics** |
| Abiraterone | Abiraterone inhibits the CYP17A1 enzyme, essential for androgen production. | mCRPC (COU-AA-301, COU-AA-302); mHSPC (LATITUDE, STAPEDE) | Fluid retention, hypokalemia, and hypertension | Concomitant corticosteroid use is essential to mitigate AEs from reduced adrenal corticosteroid production. |
| Enzalutamide | They act by selectively antagonizing the AR, blocking its nuclear translocation, co activator recruitment, and DNA binding. | mCRPC (AFFIRM, PREVAIL); mHSPC (ARCHES, ENZAMET); nmCRPC (PROSPER) | Dizziness, headache, seizures, among other neurological symptoms. | An early classic novel AR antagonist, it is currently approved for the broadest range of uses in advanced prostate cancer. |
| Apalutamide |  | mHSPC (TITAN); nmCRPC (SPARTAN) | Rash, pruritus, hypothyroidism | High antiandrogenic efficacy and the ability to inhibit AR overexpression or the AR F877L mutation. |
| Darolutamide |  | nmCRPC (ARAMIS); mHSPC (ARASENS) | Cardiac arrhythmia, heart failure, and coronary-artery disorder | High antiandrogenic activity and the ability to inhibit AR T877A, W742L, and F876L mutations. |
| Rezvilutamide |  | mHSPC (CHART) | Hepatotoxicity | Suited for mHSPC patients with high tumor burden. |

RCT: randomized clinical trial; AE: adverse effect; AR: androgen receptor; CYP17A1: cytochrome P450 17A1; mCRPC: metastatic castration-resistant prostate cancer; mHSPC: metastatic hormone-sensitive prostate cancer; nmCRPC: non-metastatic castration-resistant prostate cancer
